# Bilinguals on the footbridge: the role of foreign-language proficiency in moral decision making

**DOI:** 10.1017/S1366728924000312

**Published:** 2024-04-25

**Authors:** Federico Teitelbaum Dorfman, Boris Kogan, Pablo Barttfeld, Adolfo M. García

**Affiliations:** 1 Cognitive Neuroscience Center, Universidad de San Andrés, Buenos Aires, Argentina;; 2 Cognitive Science Group, Facultad de Psicología, Instituto de Investigaciones Psicológicas (IIPsi, CONICET-UNC), Universidad Nacional de Córdoba, Córdoba, Argentina;; 3 National Scientific and Technical Research Council (CONICET), Buenos Aires, Argentina;; 4 Department of Philosophy, Faculty of Humanities, National University of Mar del Plata, Buenos Aires, Argentina;; 5 Global Brain Health Institute, University of California, San Francisco, CA 94158, USA and Trinity College Dublin, Dublin, Ireland; 6 Departamento de Lingüística y Literatura, Facultad de Humanidades, Universidad de Santiago de Chile, Santiago, Chile

**Keywords:** bilingualism, moral foreign-language effect, footbridge dilemma, foreign-language proficiency, modulating factors

## Abstract

Socio-cognitive research on bilinguals points to a moral foreign-language effect (MFLE), with more utilitarian choices (e.g., sacrificing someone to save more people) for moral dilemmas presented in the second language (L2) relative to the first language. Yet, inconsistent results highlight the influence of subject-level variables, including a critical underexplored factor: L2 proficiency (L2p). Here we provide a systematic review of 57 bilingualism studies on moral dilemmas, showing that L2p rarely modulates responses to impersonal dilemmas, but it does impact personal dilemmas (with MFLEs proving consistent at intermediate L2p levels but unsystematic at high L2p levels). We propose an empirico-theoretical framework to conceptualize such patterns, highlighting the impact of L2p on four affective mediating factors: mental imagery, inhibitory control, prosocial behavior and numerical processing. Finally, we outline core challenges for the field. These insights open new avenues at the crossing of bilingualism and social cognition research.

## 1. Introduction

Moral cognition is a multidimensional neurocognitive domain implicated in decisions, judgments and inferences about what constitutes required or acceptable social behavior (Reese et al., 2020; Van Bavel et al., 2015; Wong, 2019; Yu et al., 2019). Its deployment in daily life involves reasoning, impulse control, experience learning and conceptualizations of socially relevant values, traits and events (Greene, 2015). Such mechanisms can be influenced by bilingual experience (Costa & Sebastián-Gallés, 2014; Titone & Tiv, 2022), prompting the prediction that moral decisions may change depending on whether scenarios are presented in the participants’ first or second language (L1, L2). Yet, this moral foreign-language effect (MFLE) has been only inconsistently observed (Brouwer, 2021; Čavar & Tytus, 2018; Dylman & Champoux-Larsson, 2020; Winskel & Bhatt, 2020), suggesting that it may be affected by interindividual variability. Here we tackle the issue focusing on L2 proficiency (L2p, a person’s current level of mastery of her or his L2), a factor that varies widely among bilinguals, has been measured in most MFLE studies, and systematically influences outcomes in relevant domains. Examining this topic is vital to illuminate the links between linguistic experience and moral cognition, constrain models of socio-affective processing in bilinguals and inform translational developments therefrom.

Most evidence on the MFLE comes from moral decision tasks. These place participants in a first-person position, face them with a moral dilemma and require them to make a choice that will be beneficial for some people but detrimental (often deadly) to others (Bartels et al., 2015; Tassy et al., 2013; Yu et al., 2019). The field has favored incongruent moral dilemmas, presenting a utilitarian option that maximizes aggregate welfare (e.g., letting one person die to save another five) and a deontological option based on moral norms (e.g., inhibiting action to save one person at the expense of other five) (Conway & Gawronski, 2013).^1^

These can be divided into impersonal dilemmas, in which utilitarian decisions involve no physical contact with the victim; and personal dilemmas, in which such decisions require direct use of force on the victim (Greene, 2014). The former include the trolley or switch dilemma (Thomson, 1985), where a trolley fast approaches a group of people on the rails and only the participant can press a switch that changes the trolley’s direction, sacrificing a single person instead of multiple ones. On the other hand, a typical personal version of this task would be the footbridge dilemma (Thomson, 1976), where the participant, witnessing the scene from a footbridge, can save five lives by pushing another person in front of the vehicle.

A foundational study on bilinguals (Costa et al., 2014b) reported more utilitarian choices when dilemmas were presented in L2 as opposed to L1, extending evidence of reduced intuitive biases during L2 processing (Costa et al., 2014a; Keysar et al., 2012). This has sparked the notion that bilinguals’ moral cognition depends on the language used, arguably due to a combination of linguistic, executive and affective factors (Hayakawa et al., 2016; Pavlenko, 2017). Yet, ulterior evidence proved mixed, with only some studies replicating this finding and meta-analytical results revealing only small (Del Maschio et al., 2022a) or small-to-moderate (Circi et al., 2021; Stankovic et al., 2022) MFLEs. Results are inconsistent even for the footbridge dilemma, the task offering the strongest supporting evidence (Circi et al., 2021; Del Maschio et al., 2022a; Stankovic et al., 2022). Such heterogeneity indicates that the effect may be modulated by subject-level variables impinging on bilingual cognition, crucially including L2p.

L2p represents an individual’s degree of L2 knowledge and skills to function in specific communicative situations and modalities (Hulstijn, 2011). The construct encompasses multiple subfactors, including productive and receptive abilities across phonological, lexico-semantic, morphosyntactic and pragmatic factors (Gullifer et al., 2021; Olson, 2023). L2p ranks among the most widely studied individual variables in bilingualism research (Olson, 2023; Park et al., 2022), be it as a cut-off variable (for sample selection), as a controlled variable (for group matching) or as a manipulated variable (for testing its impact on specific outcome measures) (Hulstijn, 2012; Olson, 2023). It can be measured with objective methods (e.g., standardized tests, experimental tasks) or via subjective ratings (e.g., self-report questionnaires). The latter are dominant in the literature, accounting for more than 60% of published studies (Olson, 2023). The same is true for MFLE studies – in fact, 86% of those considered in this work used subjective measures exclusively (see the Supplementary material).

Based on conventional or sample-specific cut-offs, a distinction can be made between bilinguals with low, intermediate and high L2p, among other subdivisions. For example, studies using the Language Experience and Proficiency Questionnaire (e.g., Kaushanskaya et al., 2020; Marian et al., 2007) typically establish a cut-off of 7 (out of 10) to establish high L2p, while other works, including MFLE research (Corey et al., 2017; Costa et al., 2014b; Geipel et al., 2015), split participants into low and high L2p groups based on the sample’s median L2p – usually around 70% on a 0–100% scale. Importantly, although framing L2p as a continuum offers powerful avenues for correlational research, its discretization through cut-offs offers a useful heuristic given the often subtle nature of MFLEs.

Regardless of its measurement, this variable is known to modulate diverse cognitive domains. As such, higher L2p levels have been linked to heightened emotional processing (activation of affective mechanisms by arousing stimuli; Caldwell-Harris, 2015; Harris et al., 2006; Imbault et al., 2021; Pavlenko, 2017; Sutton et al., 2007), enriched mental imagery (visual or otherwise perceptual representations of events in the absence of direct sensory input; Hayakawa & Keysar, 2018), increased inhibitory control (the capacity to suppress prepotent information to favor adequate task completion; Goral et al., 2015; Hui et al., 2020; Thanissery et al., 2020), more efficient lexico-semantic processing (access to and retrieval of words’ meanings; Abutalebi, 2008; Bialystok & Craik, 2010; Cuppini et al., 2013; Dijkstra et al., 2019; Ibáñez et al., 2010; Keating, 2017; Liberto et al., 2021; Zheng et al., 2020), stronger embodied resonance (reactivation of sensorimotor brain mechanisms subserving the bodily experiences denoted by linguistic material; Bergen et al., 2010; Birba et al., 2020; Ibáñez et al., 2010; Kogan et al., 2020; Vukovic, 2013), enhanced code switching flexibility (alternation between languages during continuous speech; Kootstra et al., 2012) and better numerical processing (the ability to perform mental operations involving digits and figures; Hoshino et al., 2010; Van Rinsveld et al., 2016). Higher L2p also impacts complex social phenomena, as it is related to more effective lying and lie detection (Caldwell-Harris & Ayçiçeǧi-Dinn, 2009; Elliott & Leach, 2016), increased prosocial sentiments (Miller et al., 2021), greater altruism (Liu et al., 2022) and enhanced theory of mind capabilities (Nguyen & Astington, 2014). Briefly, L2p is a key determinant of multiple operations in bilingual cognition.

Suggestively, the domains abovementioned are critically engaged during moral dilemma tasks. Consider the footbridge dilemma. In deciding whether to push the man or not, participants must tap into emotional processes (as affective reactions are commonly found on sacrificial dilemmas) (Chan et al., 2016; Klenk, 2021), lexico-semantic processing (as conceptual information must be accessed to understand and perform the task), mental imagery (as the scene is either explicitly or implicitly visualized) (Hayakawa & Keysar, 2018), action inhibition (as prepotent decisions may need to be suppressed for moral reasons) (Gawronski et al., 2017), embodied resonance (as the notion of pushing the man likely engages sensorimotor simulations) (García et al., 2019; Greene, 2014) and numerical processing (as the number of people to “exchange” for a single life is a relevant decision factor) (Cao et al., 2017). L2p may impact the MFLE by modulating these processes. Indeed, during L2 tasks, low L2p reduces the vividness and sensorimotor reactivations of mental scenes (Altın et al., 2022; Birba et al., 2020), hampers inhibitory reactions (Hui et al., 2020; Thanissery et al., 2020), lessens prosocial and empathic tendencies (Dewaele & Wei, 2012; Ferré et al., 2022) and limits numerical processing capacities (Garcia et al., 2021; Van Rinsveld et al., 2016). Accordingly, L2p could partly account for the heterogeneous results around the MFLE.

Some studies and reviews have tackled this hypothesis by design (Brouwer, 2019, 2021; Čavar & Tytus, 2018; Circi et al., 2021; Costa et al., 2014b; Del Maschio et al., 2022a; Geipel et al., 2015; Hayakawa & Keysar, 2018; Hayakawa et al., 2017; Shin & Kim, 2017), while several others have acknowledged such a link, albeit briefly (Costa et al., 2019; Driver, 2020; Dylman & Champoux-Larsson, 2020; Hayakawa et al., 2016; Miozzo et al., 2020; Pavlenko, 2017; Winskel & Bhatt, 2020; Wong & Ng, 2018). Moreover, meta-analytical evidence underscores correlations between self-reported L2p and the MFLE on personal dilemmas (Stankovic et al., 2022). However, the literature lacks a systematic conceptual framework describing the multidimensional impact that L2p could exert on the MFLE. Some studies have factored it out, and roughly half the corpus considers it only for group-matching purposes. Similarly, most reviews address it only vaguely amidst several other potential subject-level confounds – an issue that is further complicated by the lack of standardized L2p measures across studies (Hulstijn, 2011; Tomoschuk et al., 2019; Zell & Krizan, 2014). Furthermore, these issues impinge on meta-analyses of the MFLE, particularly within the two that found no significant L2p modulations – pointing at measurement heterogeneity across the literature and low statistical power (Circi et al., 2021; Del Maschio et al., 2022a). Crucially, too, a detailed rationale is lacking of how L2p might modulate multiple processes recruited during moral decision making, distancing the field from overarching accounts of the construct. In fact, no integrative work has focused at length on L2p as a potential modulator of the MFLE. Thus, an important gap emerges toward understanding how this intraindividual factor may impinge on core interindividual phenomena across bilingual persons.

Here, we propose an empirico-theoretical framework to conceptualize the impact of L2p on moral decision making. First, we provide a systematic review of bilingualism studies on incongruent moral dilemmas. Then, we distill the main findings regarding MFLE in bilinguals with (a) intermediate and (b) high L2p. Third, we provide a rationale for interpreting how L2p might account for the observed patterns due to its influence on multiple task-relevant factors. Finally, we outline core challenges and opportunities for future research. Overall, we aim to lay the groundwork for strategic examinations of how bilingual experience may shape a fundamental aspect of daily social cognition.

## 2. Review criteria

**Figure 1.**
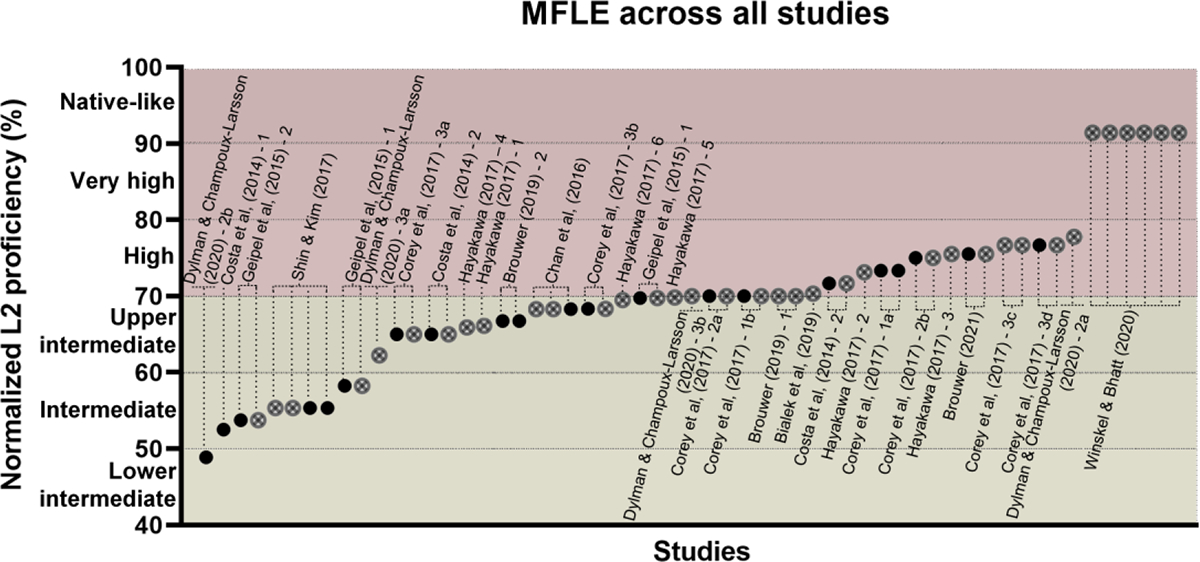
Detailed pipeline for identification, screening and selection of reports, based on PRISMA guidelines (Page et al., 2021). The asterisk (*) denotes citations in identified papers and additional web searches. L2p: foreign-language proficiency.

The review was performed in accordance with the Preferred Reporting Items for Systematic Reviews and Meta-analysis (PRISMA) guideline (Page et al., 2021). Articles were retrieved via ScienceDirect (www.sciencedirect.com), PubMed (www.pubmed.ncbi.nlm.nih.gov), the Web of Science (www.webofscience.com) and Google Scholar (www.scholar.google.com), with a final search completed in December 2021 (Figure 1). Searches were done with the term “foreign language effect” alone as well as with the following combinations: “foreign language effect” OR “foreign language” OR “bilingual” AND “moral” OR “decisions” OR “dilemma” OR “emotions” OR “empathy.” The same terms along with the term “review” were introduced on Google’s main search engine to detect papers absent in the online libraries. Additionally, the terms “moral,” “decisions,” “dilemma,” “emotions,” and “footbridge” were checked on the *Bilingualism: Language and Cognition* website. Three recent meta-analyses were also revised (Circi et al., 2021; Del Maschio et al., 2022a; Stankovic et al., 2022). Finally, the References sections of all papers, including five reviews, were screened for further relevant publications.

The above process resulted in 57 papers. First, out of 74 screened records, we excluded those that did not elicit decisions on moral dilemmas (e.g., those involving economic/framing dilemmas, such as the Asian disease problem, *n* = 43) and/or did not report original experiments (e.g., reviews, *n* = 6). We further excluded non-peer-reviewed articles (*n* = 3), as these lack fundamental checks of scientific quality and may report findings that deviate from those found in ulterior peer-reviewed versions, although we checked for discrepant evidence to account for potential publication bias effects. Application of such criteria led to 22 articles. These were screened for inclusion considering the following parameters: (i) presence of at least one group of bilingual participants, (ii) inclusion of at least one task involving decisions about one or more incongruent moral dilemmas (i.e., those involving a first person moral “yes or no” decision), (iii) use of statistical tests on the presence or absence of an MFLE and (iv) reports of mean L2p based on a Likert-type scale. This resulted in the exclusion of nine articles, which (a) failed to report L2p values (*n* = 4), (b) framed the use of regionalisms as L2 use (*n* = 1) or (c) quantified L2p via standardized exams or basic tasks that did not allow for normalization with the standard Likert scales used to measure L2p in most studies (*n* = 6). The latter exclusion criterion was applied because Likert-based self-reports of L2p (a) represent the most common measure of the construct (Hulstijn, 2012), maximizing comparability of present conclusions with relevant literature; (b) constitute good predictors of objective proficiency (Gollan et al., 2012; Langdon et al., 2005; Marian et al., 2007) and (c) allow for a linear normalization of outcomes to reveal potential proficiency-related modulations of the MFLE.

Our final database included 11 articles spanning 57 experiments (see the Supplementary material). These were systematically analyzed on a spreadsheet containing columns for the following aspects: title, authors, year of publication, number of participants, mean age, L1 and L2 of the sample(s), L2p (including method of measurement and reported value, if applicable), age of L2 appropriation, experimental stimuli, types of dilemma involved (personal/impersonal), results regarding the MFLE and additional relevant results. Clarification for data from one paper was required via an e-mail to its corresponding author, given that it seemed to contain erroneous information (lower L1p than L2p scores). For details, see the Supplementary material (Table S1).

## 3. Literature overview

**Figure 2.**
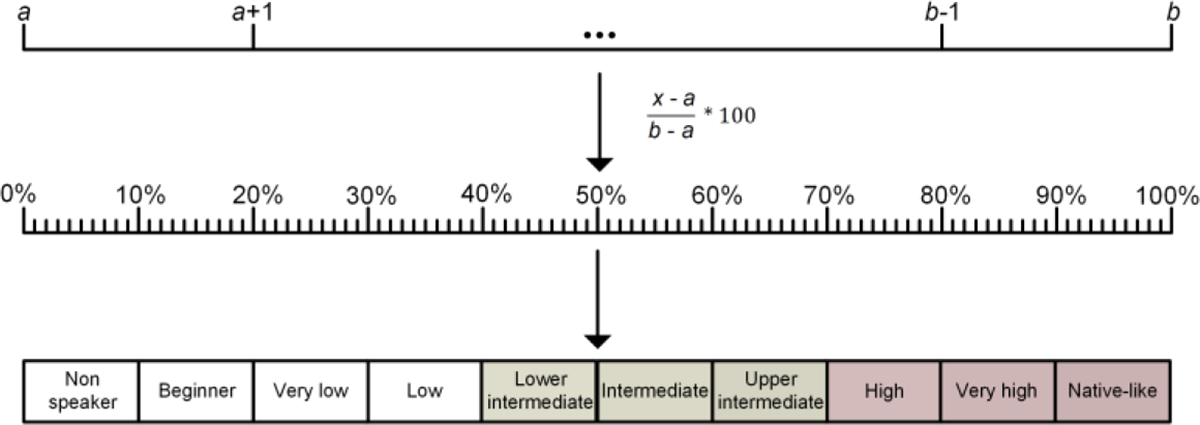
L2p normalization formula. *x* is the reported L2p mean and *a* and *b* represent the minimum and maximum values of the scale, respectively. The normalization formula offers percent values for each average L2p. Finally, the percent scale values are classified between ten different qualitative levels to ease their descriptive analysis. Intermediate and high L2p levels are distinguished in color.

**Figure 3.**
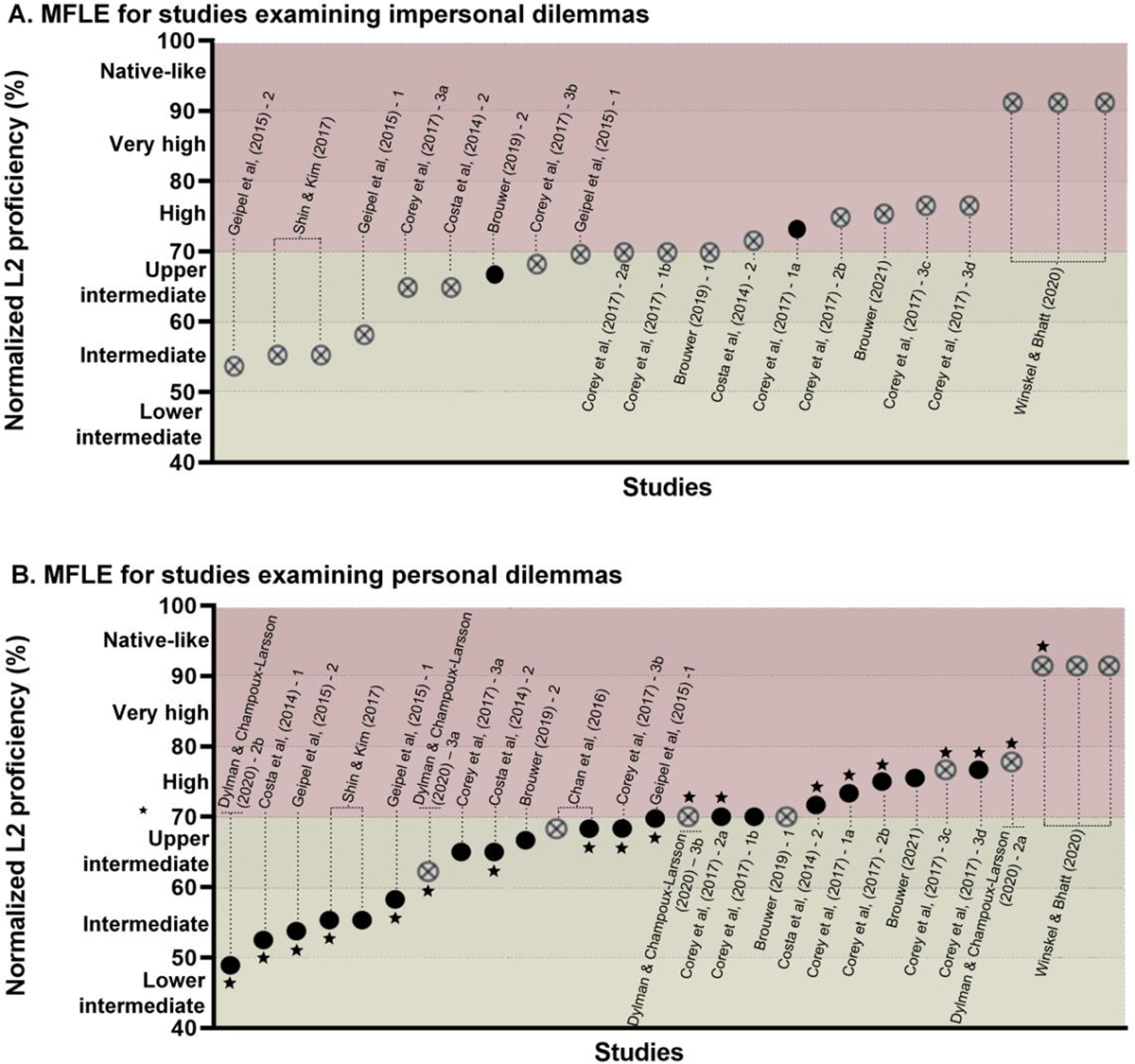
MFLE on impersonal (A) and personal (B) dilemmas. Studies are sorted from left to right on the *X* axis based on their samples’ normalized L2p level. Black circles (●) denote significant MFLEs. Crossed circles (⊗) denote non-significant MFLEs. Stars (★) indicate that the experiment used solely the footbridge dilemma. Only studies that exclusively distinguish personal and impersonal dilemmas are included; a figure with all studies included in the review can be found in the Supplementary material (Figure S1).

Experiments were organized based on the participants’ Likert-based L2p estimations. These were derived from task-relevant macroskills (reading or listening) when available, since test modality might influence relevant L2p skills differentially (Hulstijn, 2011; McLean et al., 2020; Wagner, 2013) and MFLE meta-analyses have found significant L2p effects only when differentially analyzing reading and listening L2p (Stankovic et al., 2022) – as opposed to average global L2p measures (Circi et al., 2021; Del Maschio et al., 2022a). Ratings of global proficiency were considered only when such task-relevant results were not reported. Since the corpus encompassed different scale ranges, L2p ratings were normalized to ensure comparability across experiments. Each Likert scale was framed as a continuous variable from 0% to 100%, encompassing ten qualitative L2p levels (Figure 2). Each L2p mean value was normalized following a reported formula (Del Maschio et al., 2022a): ((*x* − *a*)/(*b* − *a*)) × 100, where *x* is the reported L2p mean and *a* and *b* represent the minimum and maximum values of the scale, respectively. Studies were then sorted based on their samples’ normalized L2p value, identifying those that yielded significant and non-significant MFLEs in impersonal and personal dilemmas (Figure 3). All plots start from the lower intermediate L2p level, as no lower proficiency studies were included for review.

Considering the patterns in the figures above, together with compatible meta-analytical evidence (Stankovic et al., 2022), studies are next reviewed for each of those L2p levels separately, yielding 28 experiments with intermediate L2p levels (<70% normalized L2p) and 29 high L2p levels (≥70% normalized L2p).

Finally, identification of mediating domains for our explanatory framework was also based on literature-driven criteria. Specifically, domains were deemed relevant if at least two MFLE studies implicated them in decision-making patterns, and if they were related to L2p in at least one further study from the general bilingualism literature.

### 3.1. The MFLE at intermediate L2p levels

**Figure 4.**
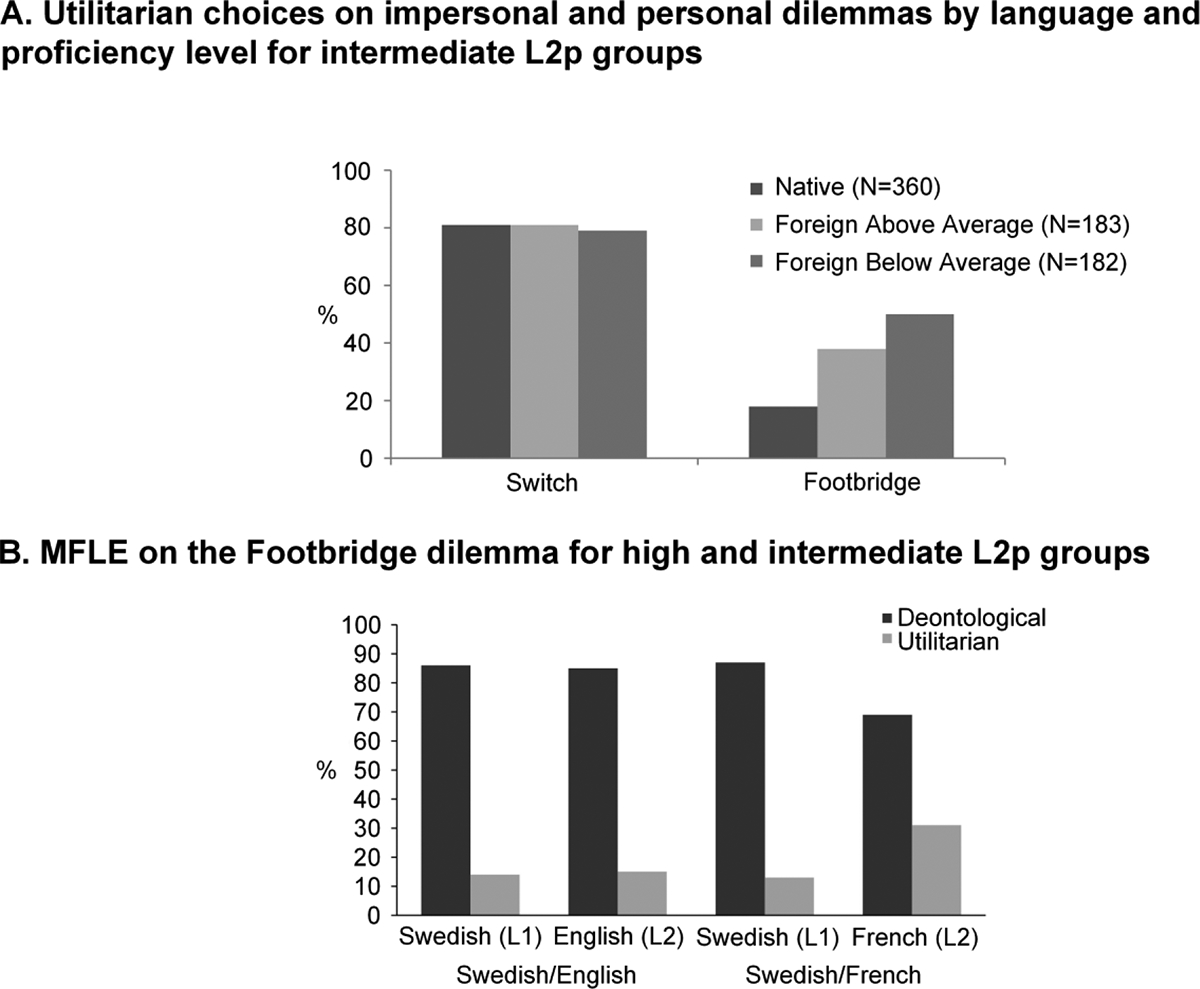
Outstanding results showing the role of L2p on impersonal and personal moral dilemmas. (A) Utilitarian choices resulting from a moral decision task with an impersonal (trolley) and a personal (footbridge) dilemma, showcasing an MFLE only on the personal one. Divided in above average and below average groups of self-rated L2p, the MFLE seems stronger on the lower L2p subjects. (B) Results from a footbridge dilemma task on two groups with different L2 and different L2p levels. The Swedish–English group had a high normalized L2p = 77.77% and failed to show an MFLE. The Swedish–French group had an intermediate normalized L2p = 48.88% and showed a significant increase on utilitarian choices for L2 responses. Panel A: reprinted from PLoS ONE 9(4): e94842, by Albert Costa, Alice Foucart, Sayuri Hayakawa, Melina Aparici, Jose Apesteguia, Joy Heafner and Boaz Keysar, “Your Morals Depend on Language” (open access), Copyright 2014, https://doi.org/10.1371/journal.pone.0094842. Authorized reproduction under the terms of the Creative Commons Attribution License. Panel B: reprinted from Cognition, Volume 196, by Alexandra S. Dylman and Marie-France Champoux-Larsson, “It’s (not) all Greek to me: Boundaries of the foreign language effect,” 104148, Copyright (2020), with permission from Elsevier.

*Impersonal dilemmas* consistently exhibit more utilitarian than non-utilitarian choices across studies. Yet, this pattern does not differ between the participants’ two languages. This is true for the widely used switch/trolley dilemma, which yielded similar response patterns in both languages when based either on its typical question “Would you push the man?” (Corey et al., 2017; Costa et al., 2014b; Geipel et al., 2015; Shin & Kim, 2017; Figure 4A), or on an outcome-driven paraphrasis such as “Would you let five people die?” (Corey et al., 2017). These results are robust irrespective of the participants’ specific L1s and L2s (Costa et al., 2014b; Geipel et al., 2015). Yet, most studies on impersonal dilemmas had English as their L2, inviting further research on more diverse language pairs – a critical point given that overreliance on English has been shown to bias findings in related fields (Blasi et al., 2022; García et al., 2023).

A non-significant MFLE was also observed in the fumes dilemma (Shin & Kim, 2017) which requires deciding whether toxic gas threatening three patients at a hospital should be redirected by pressing a switch, killing only one patient in another room (Greene et al., 2008). Moreover, non-significant effects were observed in studies comparing L2p subgroups or performing correlations between L2p and response type (Corey et al., 2017; Costa et al., 2014b; Geipel et al., 2015; Shin & Kim, 2017) – except in Corey et al. (2017), experiment 3a, where a significant negative correlation between L2p and odds of making an utilitarian choice was found for a classic written switch dilemma. The only partial exception comes from Brouwer (2019), experiment 2, who observed a significant MFLE across six dilemmas (three impersonal, three personal), with no interaction between language and dilemma type. Suggestively, this is the only experiment with this L2p level in the corpus that used auditory stimuli. This point is noteworthy because auditory input can attenuate emotional responses during L1 (but not L2) processing, potentially prompting differential moral response patterns in each language (Jankowiak & Korpal, 2018).

A different pattern emerges with *personal dilemmas*, which reveal consistent MFLEs across studies. At intermediate L2p levels, utilitarian choices are significantly more frequent in L2 than in L1. This was observed for English–Spanish (Costa et al., 2014b, experiment 2; Figure 4A), Chinese–English (Chan et al., 2016, footbridge only; Geipel et al., 2015, experiment 2), Spanish–English (Corey et al., 2017, experiments 3a and 3b), Korean–English (Shin & Kim, 2017), Italian–English (Geipel et al., 2015, experiment 1), Dutch–English (Brouwer, 2019, 2021), Swedish–French (Dylman & Champoux-Larsson, 2020, experiment 2b; Figure 4B) and Italian–German (Geipel et al., 2015, experiment 1) bilinguals. The effect is most systematic for the footbridge dilemma in its classical version (Chan et al., 2016; Costa et al., 2014b; Dylman & Champoux-Larsson, 2020, experiment 2b; Geipel et al., 2015; Shin & Kim, 2017), even when comparing samples with different languages as L1 and L2 (Costa et al., 2014b; Geipel et al., 2015).

The MFLE on the footbridge dilemma was replicated when participants are given the option to sacrifice the man by pushing a button, baring physical brute force but maintaining the instrumental nature of the death – a modification that seemed to reduce the magnitude of the effect (Corey et al., 2017, experiment 3a). An MFLE was also observed when the question is changed from “Would you push the man?” to “Would you let five people die?,” suggesting that it is robust even when consequences are highlighted (Corey et al., 2017, experiment 3b). Significant MFLEs also emerge in other personal dilemmas (involving, e.g., decisions on suffocating a baby to save more people, and transplanting organs from a healthy patient to save other five) with both written (Shin & Kim, 2017) and auditory (Brouwer, 2019) stimuli. Reinforcing these patterns, the frequency of utilitarian responses in L2 was shown to be higher for subgroups with lower L2p and to correlate negatively with L2p (Costa et al., 2014b; Geipel et al., 2015; Shin & Kim, 2017; Figure 4A) – although no such correlations emerged in the Corey et al. (2017) modified footbridge dilemmas (experiments 3a and 3b).

A noteworthy exception can be found in Chan et al. (2016), who failed to find an MFLE in analyses performed over 22 personal dilemmas. However, this study did find a significant MFLE when isolating responses to the footbridge dilemma. Interestingly, the MFLE in personal dilemmas seems to disappear when the participants’ two languages are structurally or typologically similar, as seen for Swedish–Norwegian and high L2p Norwegian–Swedish bilinguals on the footbridge dilemma (Dylman & Champoux-Larsson, 2020, experiments 3a and 3b),^2^ and the same lack of MFLE was found on moral choice decision tasks disregarding type for German–English and English–German bilinguals (Hayakawa et al., 2017, experiments 1, 4, 5 and 6).

Overall, research on the MFLE at intermediate L2p levels reveals three tentative patterns. First, this effect seems mostly absent for impersonal dilemmas, as seen in roughly 80% of experiments. Second, it proves quite systematic for personal dilemmas, as seen in nearly 85% of experiments (especially those using the footbridge dilemma, yielding significant MFLEs in 90% of cases). Third, utilitarian decisions in L2 seem to increase as L2p decreases. Finally, the few exceptions to these patterns might be related to presentation modality and language similarity. These observations are discussed in section 4.

### 3.2. The MFLE at high L2p levels

As observed for mid-proficiency bilinguals, *impersonal dilemmas* also yield non-significant MFLEs in high L2p groups. Most studies examined the switch/trolley dilemma, all but one reporting non-significant MFLEs across written (Brouwer, 2019, 2021; Corey et al., 2017; Costa et al., 2014b; Dylman & Champoux-Larsson, 2020) and auditory (Brouwer, 2021) modalities, even for native-like L2p participants (Winskel & Bhatt, 2020). Null effects were also reported upon switching languages between dilemma types, adding social identification factors or highlighting consequences and responsibilities on the action (Corey et al., 2017). The same occurred with other impersonal dilemmas requiring participants to decide whether to keep the money upon finding a wallet, lie on their tax returns (Brouwer, 2019, 2021), choose who should lose the prize money on a TV show (Winskel & Bhatt, 2020). Likewise, a meta-analysis of Corey et al. (2017) experiments showed no effects of L2p or language on choices for the switch dilemma overall – the only exception was experiment 1a, in which the odds of utilitarian choices on a regular switch dilemma increased for the L2 group, though less significantly than or the footbridge dilemma. Also, in Corey et al.’s (2017) study, only two out of its eight experiments yielded significant differences between higher and lower L2p subgroups, the former making more utilitarian choices on the switch dilemma.

*Personal dilemmas*, on the other hand, did not yield the same results observed for intermediate L2p levels. Far from consistent, MFLEs were less common across high L2p groups. Four experiments failed to find MFLE on the footbridge dilemma. This happened in highly proficient Hindi–English bilinguals (Winskel & Bhatt, 2020), and in experiments 2a and 3b of Dylman and Champoux-Larsson (2020), involving Swedish–English (with very high L2p; Figure 4B) and Norwegian–Swedish bilinguals, respectively. Notably, a non-significant MFLE was found when accounting for aversion by changing the question to “Would you let five people die by not pushing him?” (Corey et al., 2017, experiment 3c). On the other hand, the classic written version of the footbridge dilemma did yield an MFLE in high L2p Spanish–English participants (Corey et al., 2017, experiments 1a and 2a; Costa et al., 2014b, experiment 2). The same occurred when the task made explicit the victims’ nationalities (to test for social group identification) and when the utilitarian decision caused the man to be disabled for life instead of killing him (Corey et al., 2017, experiments 2b and 3d).^3^

The only report that checked for L2p as a possible mediator of the MFLE on personal dilemmas was Corey et al. (2017). Intra-experimental results were less conclusive, since experiments 1b, 2a, 2b and 3d failed to find differences, while only two (1a and 3c) found evidence of low L2p groups making more utilitarian choices than high L2p and L1 groups on the footbridge dilemma. Yet, a meta-analysis of all experiments in the study found that utilitarian choices on the footbridge dilemma increased as L2p decreased.

The inconsistency of the MFLE in personal dilemmas is not exclusive to the footbridge task. A non-significant MFLE was found in native-like Hindi–English bilinguals on two personal dilemmas in which direct actions on a TV show determined whether a family or a player would fall or be pushed into the water and lose all their prize money (Winskel & Bhatt, 2020). Likewise, no MFLE was observed in Dutch–English bilinguals across several personal dilemmas (Brouwer, 2019). Contrastingly, a significant MFLE did emerge on a similar task and with a similar sample upon aggregating the results of the footbridge and other dilemmas in both written and auditory modalities (Brouwer, 2021). A significant MFLE was also found on Spanish–English bilinguals for the terrorist dilemma, in which deciding to kill a terrorism hostage entails saving other five people (Corey et al., 2017, experiment 1b).

Lastly, note that a battery of 24 moral dilemmas without personal/impersonal classifications also failed to yield evidence of an MFLE on Polish L1 speakers with either English, German, Spanish or French as their L2 (Białek et al., 2019). This study, as the one by Hayakawa et al. (2017), only found evidence of an MFLE when checking for more complex parameters than the utilitarian versus deontological distinction, as those provided by process-dissociation paradigms (Conway & Gawronski, 2013) or the consequences, norms and preference for inaction (CNI) model (Gawronski et al., 2017; Hennig & Hütter, 2021). Different accounts looking to complexify the MFLE will be addressed in section 4.

Finally, we checked three non-peer-reviewed MFLE articles for discrepant evidence to account for potential publication bias effects. Only one met our exclusion criteria, reporting a null MFLE for the footbridge dilemma across high L2p bilinguals, alongside and non-significant tendency toward an MFLE in intermediate L2p bilinguals (Zeybek, 2021).

Overall, across high L2p groups, impersonal moral dilemmas usually will yield non-significant MFLEs. Conversely, personal dilemmas yield unsystematic results, with half the experiments reporting non-significant MFLEs, even for the footbridge dilemma (44.4% non-significant MFLE). This pattern differs from that observed in intermediate L2p groups, who exhibited significant MFLEs in almost all personal dilemmas, especially the footbridge one. As discussed below, these patterns may be driven by numerous factors, including the self-reported nature of L2p levels, dilemma types and measurement methods.

## 4. Discussion

This systematic review examined the impact of L2p on the MFLE. Briefly, L2p rarely modulates responses to impersonal dilemmas, which typically yield non-significant MFLEs. Conversely, it does seem to impact personal dilemmas, with MFLEs proving consistent at intermediate L2p levels but unsystematic at high L2p levels. Below we discuss these findings, advance a multidimensional framework of the phenomenon and identify core challenges for the field.

The MFLE is systematically absent in impersonal dilemmas. The only exceptions correspond to a mild MFLE on the switch dilemma in Corey et al. (2017, experiment 1a) – though seven other impersonal dilemma experiments in the same report failed to find it – and to a battery of three auditory dilemmas in Brouwer (2019) – which also escaped replication in a later report (Brouwer, 2021). More crucially for our current focus, the effect remains null irrespective of L2p, as moral decisions were almost always similar between languages in both intermediate and high L2p levels. Such is the case across different dilemmas and language pairs (Corey et al., 2017; Geipel et al., 2015). This observation aligns with meta-analytic evidence (Stankovic et al., 2022) and is consistent with several reports performing L2p analyses of their experiments (Corey et al., 2017; Costa et al., 2014b; Geipel et al., 2015; Shin & Kim, 2017; Wong & Ng, 2018). Overall, impersonal dilemmas fail to yield an MFLE across varied L2p levels.

Conversely, the MFLE does seem sensitive to lower L2p in the face of personal dilemmas. Utilitarian decisions in L2 increase systematically at intermediate L2p levels, with MFLEs emerging in 85% of studies. Yet, this pattern proves inconsistent at high L2p levels, as the MFLE appears in only 50% of studies. This discrepancy seems task-independent, as the effect has proven significant for intermediate- and null for high-proficiency bilinguals on the footbridge, the baby and the vitamins dilemmas (Brouwer, 2019). Moreover, it has been reported in samples who speak typologically similar (e.g., Dutch–English; Brouwer, 2019) and typologically different (e.g., Spanish–English; Corey et al., 2017) languages. These patterns are noteworthy given that, as seen in different meta-analyses, the MFLE, at large, seems highly sensitive to task- and subject-level variables (Circi et al., 2021; Del Maschio et al., 2022a). Indeed, a recent meta-analysis found a significant negative L2p effect on utilitarian decisions when exclusively targeting personal dilemmas (Stankovic et al., 2022). This is also consistent with several reports performing L2p analyses of their experiments (Corey et al., 2017; Costa et al., 2014b; Geipel et al., 2015; Shin & Kim, 2017; Wong & Ng, 2018). Interestingly, the two intermediate L2p experiments yielding a non-significant MFLE may not have met key requisites of the hypothesis. For instance, they depicted scenarios that may not actually represent incongruent personal dilemmas – e.g., killing your grandmother in spite after she denies you a gift (Chan et al., 2016). Also, they involved highly similar languages, such as Swedish and Norwegian (Dylman & Champoux-Larsson, 2020), unlike others yielding significant MFLEs (Chan et al., 2016; Costa et al., 2014b; Shin & Kim, 2017; Winskel & Bhatt, 2020), which were based on cross-script bilinguals (Chinese, Korean, Hebrew or Hindi as L1 and English as L2). Overall, L2p seems to be a robust modulator of the MFLE in personal dilemmas.

**Figure 5.**
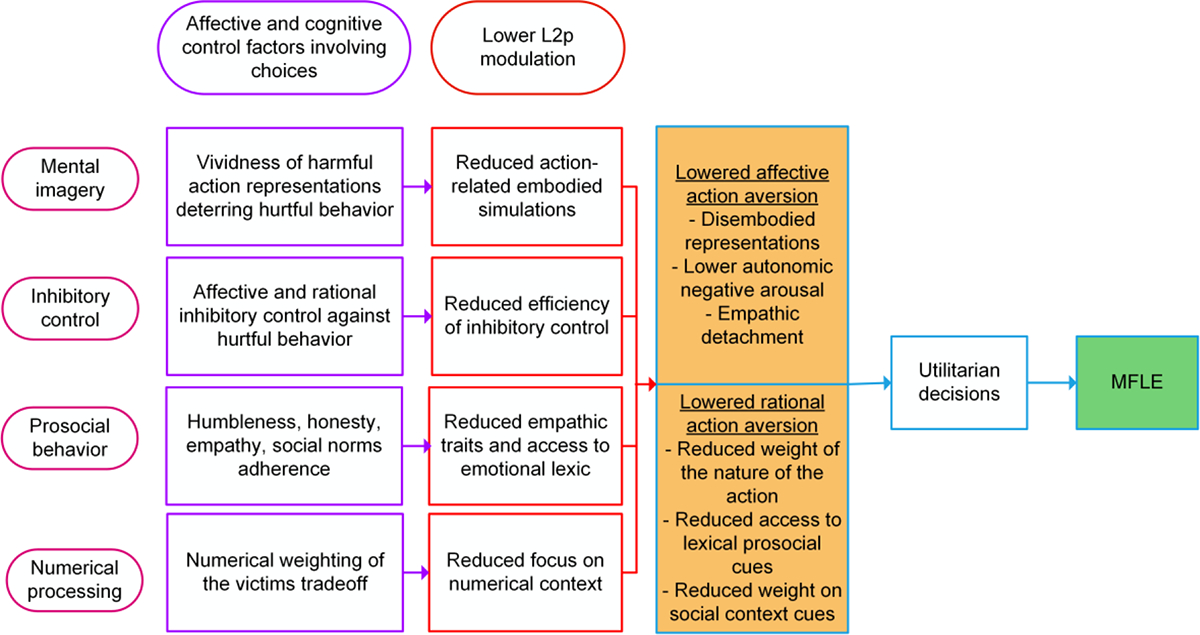
Factors mediating the impact of L2p on L2 personal moral decision tasks, leading to the MFLE. Across columns, from left to right, the figure shows (i) mediating factors, (ii) relevant affective and cognitive processes, (iii) impact of lower L2p on each process and (iv) proposed effects on action aversion. L2: second language; L2p: second language proficiency; MFLE: moral foreign-language effect.

These patterns call for a conceptual framework on the role of L2p in the MFLE. We propose that L2p influences this effect due to its influences on various domains recruited by moral cognition tasks. Four such factors would be critical in this sense, namely: mental imagery vividness, inhibitory control, prosocial tendencies and numerical processing, all analyzed under the scope of affective processing and cognitive control efforts driven by personal incongruent moral dilemmas (Figure 5). Note that, as stated in section 3, these domains were identified based on the presence of specific evidence in the literature. Accordingly, this list should not be deemed exhaustive, as the impact of L2p on the MFLE may also be shaped by other factors (e.g., level of overlap between L1 and L2 semantic systems).

Most MFLE theories account for it by reference to a classic dual decision-making system involving intuitive versus rational decisions based on fast emotional or slow normative responses, respectively (Tversky & Kahneman, 1981). The role of affection and rational deliberation as separate factors has been widely revised in the MFLE literature (Hadjichristidis et al., 2019; Hayakawa et al., 2016, 2017; Pavlenko, 2017), as discussed in section 5. Current trends highlight the role of affectivity and cognitive control when bilinguals face conflicting situations, such as incongruent moral dilemmas, and entail complex processes that are intrinsically related even at neurological levels (Inzlicht et al., 2015; Okon-Singer et al., 2015). For example, in deciding whether one person should die to save other five based on one’s direct action, individuals’ responses exhibit more negative emotional valence and arousal (Christensen et al., 2014; Tasso et al., 2017), alongside increased brain activation of emotional processing areas (Greene et al., 2001; Schaich Borg et al., 2006; Xue et al., 2013). Yet, concepts such as pure “emotion reduction” or “cognitive load excess” have been deemed too broad (McFarlane & Perez, 2020) or empirically inconclusive (Hadjichristidis et al., 2019) respectively, to be pointed as direct originators of the MFLE. Instead, it is likely that only specific processes of affectivity and cognitive control are involved in bilingual decision making on incongruent moral dilemmas. Compatibly, the presented theoretical framework will highlight four relevant factors for moral decision making on bilinguals under the scope of evidence on affectivity and cognitive control related processes, along with postulates on how low L2p might modulate them toward utilitarian choices in personal dilemmas.

### 4.1. Mental imagery

The scenarios in moral dilemma tasks evoke rich mental imagery, including conceptualizations and sensorimotor experiences associated with the situations at hand (Pearson et al., 2015). In personal dilemmas, reduced visual imaging of the intentional, instrumentalized, negative and harmful action has been proposed to increase utilitarian choices (Corey et al., 2017; Hayakawa & Keysar, 2018; Klenk, 2021). Conversely, harmful means tend to be more vivid than their beneficial ends (Amit & Greene, 2012), activating affective and prosocial deterrents of hurtful behavior.

L2p can influence the vividness of mental imagery while reading moral dilemmas. In this sense, L2p correlates positively with imagery skills (Altın et al., 2022) and with vividness of motor imaging simulations (Hayakawa & Keysar, 2018) during L2 processing. Indeed, the greater the L2p, the stronger the coupling of motor brain networks during L2 text reading, suggesting more consolidated embodied simulations that resemble L1 processing (Birba et al., 2020). Therefore, lower L2p could entail reduced sensorimotor reactivations and less vivid mental visualizations of the action on the victim, dampening affective reactions against harmful behavior. This would increase interpersonal detachment, favoring more utilitarian choices in L2 than in L1.

### 4.2. Inhibitory control

In moral (and, more particularly, personal) dilemmas, difficulties with inhibitory control – namely, the capacity to suppress ongoing thoughts, actions and emotions (Lucifora et al., 2021; Petersen et al., 2016) – can increase utilitarian decisions (Lucifora et al., 2021; van den Bos et al., 2011), likely by reducing cognitive control mechanisms that are prompted against an action/inaction choice. Indeed, inhibitory behavior toward harmful actions in personal moral decision making is likely prompted by affective aversion reported as negative arousal (McDonald et al., 2017), and it correlates with an increase in inhibition-related neurotransmitters, such as serotonin (Crockett et al., 2010; Pattij & Schoffelmeer, 2015).

L2p may affect bilinguals’ inhibitory control. In this sense, L2p is related positively with better inhibitory control in response to L2 stimuli, as seen in studies using the Simon task (Goral et al., 2015), the Stroop task (Hui et al., 2020) and other standard go/no-go inhibition tasks (Thanissery et al., 2020), likely because bilinguals have to develop stronger cognitive control systems as they process L2 stimuli more efficiently when they can inhibit and break away from L1 lexical schemas (Grant et al., 2019). Since prepotent response suppression seems critical to process stimuli that provoke stronger preferences for deontological inaction, like personal moral dilemmas (Amit & Greene, 2012; McDonald et al., 2017), a lower L2p could entail less inhibitory control on personal moral dilemmas, thus reducing affective and cognitive action deterrents and increasing utilitarian decisions.

### 4.3. Prosocial behavior

In sacrificial dilemmas, utilitarian decisions can be reduced by prosocial behavior – i.e., tendencies for positive social behavior toward others (Pfattheicher et al., 2022) – more empathic concern (Djeriouat & Trémolière, 2014; Körner et al., 2020; Takamatsu, 2018), more honest and humble personality traits (Djeriouat & Trémolière, 2014), an enhanced social context by public reveal of decisions (Andersson et al., 2020), adherence to social norms (Körner et al., 2020) and reduced psychopathic traits (Körner et al., 2020).

L2p could modulate prosocial traits in bilinguals. Notably, prosocial personality traits seem to weaken as L2p decreases. This has been shown, for example, in bilingualism studies tapping on altruism (Liu et al., 2022), prosocial amicability (Miller et al., 2021) and empathic concern (Dewaele & Wei, 2012). The evidence further suggests that higher L2p might be correlated with enhanced fast emotional reactions to social contexts (Liu et al., 2022), likely because proficient bilinguals have easier access to emotional and emotion-laden words related to socialization and cooperation (Ferré et al., 2022; Miller et al., 2021), and an empathic tendency toward learning their L2 properly (Dewaele & Wei, 2012). Social detachment as a result of bilingual experience has been often proposed as a potential explanation of the MFLE, with different works discussing how specific contexts of L2 acquisition and use could blunt emotional and normative responses (Del Maschio et al., 2022b; Hadjichristidis et al., 2019; Hayakawa et al., 2016; Miozzo et al., 2020). In this sense, L2p influences on prosociality might further modulate the MFLE. Specifically, reduced altruism and empathy in low L2p individuals might favor more interpersonally detached decisions. This would increase utilitarian choices on L2 moral decisions, potentially reflecting lower adherence to social norms (Białek et al., 2019; Hennig & Hütter, 2021) against lesser access to affective and prosocial cognitive resources.

### 4.4. Numerical processing

Numerical words shape the development and integration of numerosity skills (Leibovich et al., 2017) by evoking sensory-motor and abstract connotations of their referents (Fischer, 2018). This domain is central to moral dilemmas, which hinge heavily on quantitative estimations. Indeed, the number of potential victims when choosing not to act predicts the probability of making a utilitarian decision (Cao et al., 2017; Tassy et al., 2013). Simply put, the more potential victims the moral dilemma presents, the more likely it is to decide to push the person from the footbridge.

Suggestively, lower L2p individuals may find it harder to engage in context-sensitive quantitative processing in L2, favoring more literal and grammatical cues (Hoshino et al., 2010). Indeed, they tend to engage non-relevant grammatical L1 mechanisms when weighing numerical magnitudes in L2, which affects processing of the latter (Van Rinsveld et al., 2016), which likely occurs because L2 numerical processing in less-proficient L2 users is not as efficient as in L1, maybe even leading them to rely on L1 conceptual representations (Garcia et al., 2021). Thus, implicit estimations of the number of victims might further bias moral decisions depending on L2p. Specifically, if lower L2p reduces sensitivity to conceptual and abstract quantities, it could also interfere with weighing the contextual impact of how many people would die in the dilemma, reducing affective and prosocial reactions. This would increase the chances of utilitarian decisions, and therefore an MFLE, in mid-proficiency relative to high-proficiency bilinguals.

### 4.5. Theoretical considerations and implications

Briefly, in the realm of personal dilemmas, we posit that the impact of L2p on the MFLE would be mediated by affective and cognitive factors of at least mental imagery, inhibitory control, prosocial behavior tendencies and numerical processing.

Importantly, this view also accounts for the absence of L2p modulations, and of the MFLE at large, in impersonal dilemmas. Overall, relative to personal dilemmas, impersonal ones show no predominance of personal force (Bago et al., 2022), an increased preference for action (Corey et al., 2017; Stankovic et al., 2022) and the already discussed lack of MFLE on bilinguals. In our proposed theoretical framework, reduced L2p would modulate different factors of affectivity and cognitive control that specifically increase preference for action on personal dilemmas. Yet, impersonal dilemmas already showcase higher utilitarian rates than the ones produced by MFLE on personal ones (Corey et al., 2017; Costa et al., 2014b; Geipel et al., 2015; Stankovic et al., 2022). In impersonal dilemmas, mental imagery of the victim can prove less vivid (Amit & Greene, 2012), autonomic inhibitory reactions are reduced (McDonald et al., 2017), empathic traits exert little influence (Nasello & Triffaux, 2023) and so does the variance of number of lives (Cao et al., 2017).

Broader evidence on affectivity and cognitive control shows that, compared with personal dilemmas, impersonal ones seem to involve reduced emotional engagement (Christensen et al., 2014), less sensitivity to negative arousal states (Chan et al., 2016; McDonald et al., 2017; Wong & Ng, 2018; Youssef et al., 2012) and lower conflict processing demands (Xue et al., 2013). Overall, if these are all factors less markedly involved in L1 impersonal dilemmas, then their modulation by L2p would be negligible during L2 tasks, which would account for the absence of an MFLE in a dilemma type that already reduces affective and rational action aversion by itself.

This work carries four main implications. First, reciprocal links between linguistic and socio-cognitive skills have been reported in varied populations. For example, comprehension of social concepts correlates with the integrity of social cognition networks in neurodegenerative patients (Birba et al., 2022; Lopes da Cunha et al., 2023), and emotional language can bias moral judgments in laypersons but not in legal experts (Baez et al., 2020). Our study adds to this trend, showing that socio-affective functions may also be shaped by individual language profiles. Second, different proposals have emerged to characterize bilingual social cognition (Hayakawa et al., 2016; Pavlenko, 2017), but these have failed to systematically account for the role of L2p. The present framework partly bridges this gap, offering more nuanced views of the phenomenon while identifying specific factors to be operationalized in future research. Also, to our knowledge, this is the first MFLE review focused on the impact of L2p, offering a fine-grained view that escapes previous meta-analytical and theoretical works (Circi et al., 2021; Del Maschio et al., 2022a; Stankovic et al., 2022). Moreover, no previous work has advanced a mechanistic account of the multifactorial impact of L2p on mediators of the MFLE, let alone while including a rationale of the null MFLE typically observed in impersonal dilemmas. Third, insofar as social cognition mediates daily educational events (Li & Jeong, 2020; Sato, 2017), understanding these links could inform L2 classroom management practices. For example, depending on their students’ L2p, teachers could consider whether group activities requiring decisions from group leaders should be performed in L2 and/or supported by instructor’s facilitation. Indeed, socio-cognitive domains play increasingly prominent roles in L2 learning models (Cancienne, 2019; Miri & Pishghadam, 2021; Pishghadam et al., 2013). Also, social cognition might impact clinical decision making, inviting reflections on how to manage L2-based interactions. For instance, when bilingual caregivers are faced with decisions on a relative’s health and its impact on their family, establishing their L2p might be critical to establish which language should mediate communication with physicians – especially in cases when these do not speak the same L1 as the caregivers. Finally, since L2 research can inform public safety (Pavlenko, 2017) and educational policies (Garcia, 2017), important translational insights may be derived from systematic consideration of L2p in the field.

## 5. Outstanding challenges and future research

The evidence and the framework presented above enable new reflections on the role of L2p in moral cognition. Yet, many short-comings can be identified, paving the way for further research. Here we discuss four core challenges to be addressed in future works.

In line with more than half of studies on bilingualism (Park et al., 2022), L2p measures in our corpus are mainly restricted to subjective measures. Granted, these measures have been shown to correlate with objective outcomes and to predict behavioral performance in relevant tasks (Gollan et al., 2012; Gullifer et al., 2021; Langdon et al., 2005; Marian et al., 2007; Santilli et al., 2019). However, they are prone to self-image and desirability biases, and their results are often mis-analyzed as being normally distributed (Veríssimo, 2021). Importantly, responses to moral dilemmas might be influenced by aspects of proficiency that are often overlooked by standard instruments, such as how comfortable participants feel when using the L2 or how often they are exposed to the language. These factors might influence at least some of the modulating variables of our model (e.g., prosociality), ultimately shaping moral decision patterns and the MFLE. Future MFLE research should expand the standard operationalization of L2p to delve into these issues. We recognize that other L2p quantifications have been proposed in the literature and that participants’ classification into higher or lower L2p groups can be affected by different criteria. Future works could explore whether the MFLE patterns established here remain stable across distinct L2p quantification systems.

Also, few studies control for intercultural and interlinguistic variables when comparing multiple samples using different L1 and L2 pairs, even though proficiency ratings differ between them (Hulstijn, 2011, 2012; Tomoschuk et al., 2019). Thus, the field would greatly profit from the addition of broad, objective assessments of general and specific L2 skills. Moreover, as recently proposed (Claussenius-Kalman et al., 2021; Dewaele & Wei, 2012; Gullifer et al., 2021), future works should enrich L2p assessments with measures of interacting factors, such as daily L2 usage (Del Maschio et al., 2022b; Sulpizio et al., 2020), exposure (Gullifer et al., 2021) and L2 entropy – i.e., the balance of interactional contexts (Gullifer et al., 2021; Gullifer & Titone, 2020). This could be achieved drawing from recent models (Hulstijn, 2020; Marian & Hayakawa, 2021; Titone & Tiv, 2022) that capture the influence of contextualized individual experience (including L2p) on bilingual profiles in general, and on moral cognition in particular.

Moreover, the mediating domains we identified above are also influenced by other aspects of bilingual experience that correlate with L2p, such as age of L2 acquisition (Bialystok, 2015; Durand López, 2021; Gullifer & Titone, 2020; Kapa & Colombo, 2013), flexibility for communicative contexts of use (Gullifer et al., 2021) and L2 exposure (Anderson et al., 2020; Gullifer et al., 2021; Tomoschuk et al., 2019; Vukovic, 2013). Insofar as these use-related variables are key drivers of socio-emotional and cognitive effects in bilinguals, they may also influence the impact of L2p on the MFLE. Yet, depending on the task, the impact of L2p on different domains may be partly independent from other aspects of bilingual experience (Archila-Suerte et al., 2012; Del Maschio et al., 2022b; Oh et al., 2019; Wartenburger et al., 2003). New studies should be designed to disentangle the relative contributions of all these subject variables to the MFLE. This would allow compiling robust data on participants’ bilingual experience factors and modeling their influence on moral dilemma responses in both L1 and L2.

Other key constructs would also benefit from more refined definitions and operationalizations. For example, the notion of reduced emotional responses in L2 has been proposed as a partial explanation of the MFLE since the first report on the topic (Costa et al., 2014b). However, conceptualizations of emotional responses often overlook critical factors and fail to capture their full complexity (McFarlane & Perez, 2020), which may partly account for the mixed results regarding emotional reduction in personal moral dilemmas (Chan et al., 2016; McDonald et al., 2017; Wong & Ng, 2018; Youssef et al., 2012). Our proposed framework, and the field at large, could be enriched by more fine-grained approaches to this construct. Thorough screenings are required of culturally situated discretized emotions relevant to moral dilemmas (Michelini et al., 2019). Indeed, condensing complex emotions and emotion-laden stimuli into basic affective features such as valence and arousal risks missing cultural differences key for comparing samples (Ferré et al., 2022; Lim, 2016; Schiller et al., 2023; Yik et al., 2023). Furthermore, harmonized parameters are needed to constrain assumptions on emotional state types (McFarlane & Perez, 2020), and normative data to define emotion valence baselines between groups (McFarlane & Perez, 2020). While MFLE research mainly aims to detect increments or reductions of emotional states, several studies lack control dilemmas and general non-emotional baselines are typically absent, precluding robust comparisons even within studies.

By the same token, the standard dichotomy between a utilitarian and a deontological choice might oversimplify the processes underlying decisions in moral dilemmas. Interesting insights come from a method aimed to disentangle parameters of deontology and utilitarianism in moral dilemmas. Conway and Gawronski (2013) presented these parameters as components of a singular decision process and captured their probability of driving responses based on answers to congruent and incongruent moral dilemmas. With this approach, Hayakawa et al. (2017) found a differential reduction only on deontological responses in L2. This finding challenges MFLE accounts focused on heightened utilitarianism, as such a pattern was actually reduced in half the studies reported. Compatibly, applications of the CNI model (Gawronski et al., 2017) have revealed a distinct decrease of sensitivity to social norms in L2 moral dilemmas (Białek et al., 2019; Feng & Liu, 2022; Hennig & Hütter, 2021). Despite criticism against these models’ parameters (Baron & Goodwin, 2020; Kunnari et al., 2020), a mosaic, dimensional view of deontological and utilitarian decision could deepen our understanding of the MFLE and its links with L2p (Gawronski et al., 2020; Kroneisen & Heck, 2020; Luke & Gawronski, 2022; Zhang et al., 2018).

Utilitarianism, in particular, is often described as a moral commonsensical process that weighs welfare. Yet, it has been proposed to represent an impartial universal principle seeking maximal welfare for everyone, irrespective of personal values, closeness to the victim and gravity of consequences, among other factors (Kahane, 2015). Therefore, reports of “utilitarian choices” to sacrificial dilemmas modulated by aversion to harm others, less empathic concern or psychopathic or egotistic traits, may actually describe a proto-utilitarian principle driven by how convenient it is to cause instrumental harm (Everett & Kahane, 2020). Recent instruments (Kahane et al., 2018) allow capturing these distinctions, which may illuminate important aspects of how cognitive variables, including L2p and its modulating factors, shape moral cognition.

In this sense, it would be interesting to examine whether lower L2p differentially impacts utilitarianism in both its “negative” (permissiveness toward instrumental harm) and “positive” (impartial, universal beneficence) dimensions. These dimensions differ based on respondents’ nationality and personality (Everett et al., 2021; Navajas et al., 2021), highlighting the relevance of individual factors. L2p might be one of such variables. In particular, lower L2p would be related to lower altruism, amicability and empathic concern, which are negatively associated with instrumental harm and psychopathy (Dewaele & Wei, 2012; Everett & Kahane, 2020; Liu et al., 2022; Miller et al., 2021). Utilitarian choices could thus be increased based on the “negative” protoutilitarian principle of instrumental harm for welfare. Strategic empirical studies would be needed to test this conjecture.

Four additional points should be noted for future research. First, building on studies with native-language tasks (Crockett et al., 2017; Riva et al., 2019; Van Bavel et al., 2015), the field could incorporate neuroscientific insights, including research on the neural regions and electrophysiological mechanisms underpinning between-language differences during moral decision making. This would be crucial, for instance, to find dissociations between moral decision processes and our framework’s L2p-related modulators. Second, it would be useful to favor more naturalistic settings. Typical dilemmas allow for tight control of important variables but they are distant from the dilemmas that people face daily. If, as recently proposed, moral decisions are influenced by the plausibility of dilemmas (Carron et al., 2022; Kneer & Hannikainen, 2022; Körner et al., 2019) and participant engagement (Körner & Deutsch, 2023), then current notions about the MFLE could be enriched or even challenged by more ecological paradigms. Third, utilitarian decisions seem to increase when made by groups rather than by individuals, arguably because group increases detachment from social norms (Keshmirian et al., 2022) and from rational views in welfare discussions (Curşeu et al., 2020). Yet, no study has assessed group-level moral judgment in bilinguals, let alone focusing on L2p. This important gap should be addressed via novel designs in future research, comparing L1 performance with L2 outcomes in bilingual groups with varying L2p levels. For example, a battery of moral dilemmas (Hayakawa et al., 2017) could be presented in written form to each individual for self-completion, and then another set of comparable tasks could be administered to each group for communal discussion and consensual decision making – exclusively in L1 or in L2, respectively. This would enable comparisons between individual and group outcomes, revealing the extent to which distributed deliberation impinges on the MFLE across L2p levels. Audio recordings of the discussions could allow for automated transcription analyses to detect argumentative and otherwise communicative patterns in each group. Finally, although a systematic review was suitable for our aim of developing a theoretical framework, future works could employ complementary approaches, such as meta-analyses.

## 6. Conclusions

The MFLE seems sensitive to L2p, especially in the case of personal moral dilemmas. This effect may be mediated by mental imagery, inhibitory control, tendencies for prosocial behavior and numerical processing, all of which are sensitive to L2p. This multidimensional framework affords a synthetic explanation of diverse results in the current literature, opening rich avenues for systematic future research.

## Supplementary Material

Supplementary Material

## Data Availability

No data were used in this work other than the information retrieved from the papers reviewed, as summarized in the Supplementary material.

## Footnotes

1 Less attention has been paid to congruent dilemmas, which have yielded inconsistent results (Białek et al., 2019; Hayakawa et al., 2017; Hennig & Hütter, 2021).

2 The sample in Dylman and Champoux-Larsson (2020), experiment 3a actually has a normalized L2p = 70%, but it is included in this section for simplicity.

3 Corey et al. (2017), experiment 3e consisted on the man just being seriously injured, and no MFLE was found, but during inclusion assessments for this review, it was considered to not be an incongruent dilemma anymore so it was excluded from its scope.

## References

[R1] Abutalebi J (2008). Neural aspects of second language representation and language control. Acta Psychologica, 128(3), 466–478. 10.1016/J.ACTPSY.2008.03.014 18479667

[R2] Altın E, Okur N, Yalçın E, Eraçıkbaş AF, & Aktan-Erciyes A (2022). Relations between L2 proficiency and L1 lexical property evaluations. Frontiers in Psychology, 13, 820702. 10.3389/fpsyg.2022.820702 35369194 PMC8968421

[R3] Amit E, & Greene JD (2012). You see, the ends don’t justify the means. Psychological Science, 23(8), 861–868. 10.1177/0956797611434965 22745347

[R4] Anderson JAE, Hawrylewicz K, & Bialystok E (2020). Who is bilingual? Snapshots across the lifespan. Bilingualism: Language and Cognition, 23(5), 929–937. 10.1017/S1366728918000950

[R5] Andersson PA, Erlandsson A, Västfjäll D, & Tinghög G (2020). Prosocial and moral behavior under decision reveal in a public environment. Journal of Behavioral and Experimental Economics, 87, 101561. 10.1016/j.socec.2020.101561

[R6] Archila-Suerte P, Zevin J, Bunta F, & Hernandez AE (2012). Age of acquisition and proficiency in a second language independently influence the perception of non-native speech. Bilingualism: Language and Cognition, 15(1), 190–201. 10.1017/S1366728911000125 30197550 PMC6124681

[R7] Baez S, Patiño-Sáenz M, Martínez-Cotrina J, Aponte DM, Caicedo JC, Santamaría-García H, Pastor D, González-Gadea ML, Haissiner M, García AM, & Ibáñez A (2020). The impact of legal expertise on moral decision-making biases. Humanities and Social Sciences Communications, 7(1), 103. 10.1057/s41599-020-00595-8 38989005 PMC11230913

[R8] Bago B, Kovacs M, Protzko J, Nagy T, Kekecs Z, Palfi B, Adamkovic M, Adamus S, Albalooshi S, Albayrak-Aydemir N, Alfian IN, Alper S, Alvarez-Solas S, Alves SG, Amaya S, Andresen PK, Anjum G, Ansari D, Arriaga P, … Aczel B (2022). Situational factors shape moral judgements in the trolley dilemma in eastern, southern and western countries in a culturally diverse sample. Nature Human Behaviour, 6(6), 880–895. 10.1038/s41562-022-01319-5 35422529

[R9] Baron J, & Goodwin GP (2020). Consequences, norms, and inaction: A critical analysis. Judgment and Decision Making, 15(3), 421–442. 10.1017/S193029750000721X

[R10] Bartels DM, Bauman CW, Cushman FA, Pizarro DA, & McGraw AP (2015). Moral judgment and decision making. In Keren G, and Wu G (Eds), The Wiley Blackwell handbook of judgment and decision making (pp. 478–515). Hoboken, NJ: John Wiley & Sons, Ltd. 10.1002/9781118468333.ch17

[R11] Bergen B, Lau T-TC, Narayan S, Stojanovic D, & Wheeler K (2010). Body part representations in verbal semantics. Memory & Cognition, 38(7), 969–981. 10.3758/MC.38.7.969 20921109

[R12] Białek M, Paruzel-Czachura M, & Gawronski B (2019). Foreign language effects on moral dilemma judgments: An analysis using the CNI model. Journal of Experimental Social Psychology, 85, 103855. 10.1016/j.jesp.2019.103855

[R13] Bialystok E (2015). Bilingualism and the development of executive function: The role of attention. Child Development Perspectives, 9(2), 117. 10.1111/CDEP.12116 26019718 PMC4442091

[R14] Bialystok E, & Craik FIM (2010). Cognitive and linguistic processing in the bilingual mind. Current Directions in Psychological Science, 19(1), 19–23. 10.1177/0963721409358571

[R15] Birba A, Beltrán D, Martorell Caro M, Trevisan P, Kogan B, Sedeño L, Ibáñez A, & García AM (2020). Motor-system dynamics during naturalistic reading of action narratives in first and second language. NeuroImage, 216, 116820. 10.1016/j.neuroimage.2020.116820 32278096 PMC7412856

[R16] Birba A, Fittipaldi S, Cediel Escobar JC, Gonzalez Campo C, Legaz A, Galiani A, Díaz Rivera MN, Martorell Caro M, Alifano F, Piña-Escudero SD, Cardona JF, Neely A, Forno G, Carpinella M, Slachevsky A, Serrano C, Sedeño L, Ibáñez A, & García AM (2022). Multimodal neurocognitive markers of naturalistic discourse typify diverse neurodegenerative diseases. Cerebral Cortex, 32(16), 3377–3391. 10.1093/cercor/bhab421 34875690 PMC9376869

[R17] Blasi DE, Henrich J, Adamou E, Kemmerer D, & Majid A (2022). Over-reliance on English hinders cognitive science. Trends in Cognitive Sciences, 26(12), 1153–1170. 10.1016/j.tics.2022.09.015 36253221

[R18] Brouwer S (2019). The auditory foreign-language effect of moral decision making in highly proficient bilinguals. Journal of Multilingual and Multicultural Development, 40(10), 865–878. 10.1080/01434632.2019.1585863

[R19] Brouwer S (2021). The interplay between emotion and modality in the foreign-language effect on moral decision making. Bilingualism: Language and Cognition, 24(2), 223–230. 10.1017/S136672892000022X

[R20] Caldwell-Harris CL (2015). Emotionality differences between a native and foreign language: Implications for everyday life. Current Directions in Psychological Science, 24(3), 214–219. 10.1177/0963721414566268

[R21] Caldwell-Harris CL, & Ayçiçeǧi-Dinn A (2009). Emotion and lying in a non-native language. International Journal of Psychophysiology, 71(3), 193–204. 10.1016/j.ijpsycho.2008.09.006 18929603

[R22] Cancienne MB (2019) Embodying Macbeth: Incantation, visualization, improvisation, and characterization. In Mullen CA (Ed.), Creativity Under Duress in Education? Cham, Switzerland: Springer (pp. 361–381). 10.1007/978-3-319-90272-2_19

[R23] Cao F, Zhang J, Song L, Wang S, Miao D, & Peng J (2017). Framing effect in the trolley problem and footbridge dilemma. Psychological Reports, 120(1), 88–101. 10.1177/0033294116685866 28558527

[R24] Carron R, Blanc N, & Brigaud E (2022). Contextualizing sacrificial dilemmas within COVID-19 for the study of moral judgment. PLoS ONE, 17(8), e0273521. 10.1371/journal.pone.0273521 35994508 PMC9394814

[R25] Čavar F, & Tytus AE (2018). Moral judgement and foreign language effect: When the foreign language becomes the second language. Journal of Multilingual and Multicultural Development, 39(1), 17–28. 10.1080/01434632.2017.1304397

[R26] Chan YL, Gu X, Ng JCK, & Tse CS (2016). Effects of dilemma type, language, and emotion arousal on utilitarian vs deontological choice to moral dilemmas in Chinese–English bilinguals. Asian Journal of Social Psychology, 19(1), 55–65. 10.1111/ajsp.12123

[R27] Christensen JF, Flexas A, Calabrese M, Gut NK, Gomila A, Decety J, Van J, Stock D, & Leuven KU (2014) Moral judgment reloaded: A moral dilemma validation study. Frontiers in Psychology 5, 607. 10.3389/fpsyg.2014.00607 25071621 PMC4077230

[R28] Circi R, Gatti D, Russo V, & Vecchi T (2021). The foreign language effect on decision-making: A meta-analysis. Psychonomic Bulletin and Review, 28(4), 1131–1141. 10.3758/s13423-020-01871-z 33555512

[R29] Claussenius-Kalman H, Hernandez AE, & Li P (2021). Expertise, ecosystem, and emergentism: Dynamic developmental bilingualism. Brain and Language, 222, 105013. 10.1016/j.bandl.2021.105013 34520977

[R30] Conway P, & Gawronski B (2013). Deontological and utilitarian inclinations in moral decision making: A process dissociation approach. Journal of Personality and Social Psychology, 104(2), 216–235. 10.1037/a0031021 23276267

[R31] Corey JD, Hayakawa S, Foucart A, Aparici M, Botella J, Costa A, & Keysar B (2017). Our moral choices are foreign to us. Journal of Experimental Psychology: Learning Memory and Cognition, 43(7), 1109–1128. 10.1037/xlm0000356 28068125

[R32] Costa A, & Sebastián-Gallés N (2014). How does the bilingual experience sculpt the brain? Nature Reviews Neuroscience, 15(5), 336–345. 10.1038/nrn3709 24739788 PMC4295724

[R33] Costa A, Foucart A, Arnon I, Aparici M, & Apesteguia J (2014a). “Piensa” twice: On the foreign language effect in decision making. Cognition, 130(2), 236–254. 10.1016/j.cognition.2013.11.010 24334107

[R34] Costa A, Foucart A, Hayakawa S, Aparici M, Apesteguia J, Heafner J, & Keysar B (2014b). Your morals depend on language. PLoS ONE, 9(4), e94842. 10.1371/journal.pone.0094842 24760073 PMC3997430

[R35] Costa A, Corey JD, Hayakawa S, Aparici M, Vives ML, & Keysar B (2019). The role of intentions and outcomes in the foreign language effect on moral judgements. Quarterly Journal of Experimental Psychology, 72(1), 8–17. 10.1177/1747021817738409 30803340

[R36] Crockett MJ, Clark L, Hauser MD, & Robbins TW (2010). Serotonin selectively influences moral judgment and behavior through effects on harm aversion. Proceedings of the National Academy of Sciences of the United States of America, 107(40), 17433–17438. 10.1073/pnas.1009396107 20876101 PMC2951447

[R37] Crockett MJ, Siegel JZ, Kurth-Nelson Z, Dayan P, & Dolan RJ (2017). Moral transgressions corrupt neural representations of value. Nature Neuroscience, 20(6), 879–885. 10.1038/nn.4557 28459442 PMC5462090

[R38] Cuppini C, Magosso E, & Ursino M (2013). Learning the lexical aspects of a second language at different proficiencies: A neural computational study. Bilingualism: Language and Cognition, 16(2), 266–287. 10.1017/S1366728911000617

[R39] Curşeu PL, Fodor OC, Pavelea AA, & Meslec N (2020). “Me” versus “We” in moral dilemmas: Group composition and social influence effects on group utilitarianism. Business Ethics: A European Review, 29(4), 810–823. 10.1111/beer.12292

[R40] Del Maschio N, Crespi F, Peressotti F, Abutalebi J, & Sulpizio S (2022a). Decision-making depends on language: A meta-analysis of the foreign language effect. Bilingualism: Language and Cognition 25(4), 617–630. 10.1017/S1366728921001012

[R41] Del Maschio N, Del Mauro G, Bellini C, Abutalebi J, & Sulpizio S (2022b). Foreign to whom? Constraining the moral foreign language effect on bilinguals’ language experience. Bilingualism: Language and Cognition 14(4), 1–23. 10.1017/langcog.2022.14

[R42] Dewaele J-M, & Wei L (2012). Multilingualism, empathy and multicompetence. International Journal of Multilingualism, 9(4), 352–366. 10.1080/14790718.2012.714380

[R43] Dijkstra TON, Wahl A, Buytenhuijs F, Van Halem N, Al-Jibouri Z, De Korte M, & Rekké S (2019). Multilink: A computational model for bilingual word recognition and word translation. Bilingualism: Language and Cognition, 22(4), 657–679. 10.1017/S1366728918000287

[R44] Djeriouat H, & Trémolière B (2014). The dark triad of personality and utilitarian moral judgment: The mediating role of honesty/humility and harm/care. Personality and Individual Differences, 67, 11–16. 10.1016/j.paid.2013.12.026

[R45] Driver MY (2020). Switching codes and shifting morals: How code-switching and emotion affect moral judgment. International Journal of Bilingual Education and Bilingualism 25(3), 905–921. 10.1080/13670050.2020.1730763

[R46] Durand López EM (2021). A bilingual advantage in memory capacity: Assessing the roles of proficiency, number of languages acquired and age of acquisition. International Journal of Bilingualism, 25(3), 606–621. 10.1177/1367006920965714

[R47] Dylman AS, & Champoux-Larsson MF (2020). It’s (not) all Greek to me: Boundaries of the foreign language effect. Cognition, 196, 104148. 10.1016/j.cognition.2019.104148 31775074

[R48] Elliott E, & Leach AM (2016). You must be lying because I don’t understand you: Language proficiency and lie detection. Journal of Experimental Psychology: Applied, 22(4), 488–499. 10.1037/xap0000102 27936858

[R49] Everett JAC, & Kahane G (2020). Switching tracks? Towards a multidimensional model of utilitarian psychology. Trends in Cognitive Sciences, 24(2), 124–134. 10.1016/j.tics.2019.11.012 31911126

[R50] Everett JAC, Colombatto C, Awad E, Boggio P, Bos B, Brady WJ, Chawla M, Chituc V, Chung D, Drupp MA, Goel S, Grosskopf B, Hjorth F, Ji A, Kealoha C, Kim JS, Lin Y, Ma Y, Maréchal MA, … Crockett MJ (2021). Moral dilemmas and trust in leaders during a global health crisis. Nature Human Behaviour, 5(8), 1074–1088. 10.1038/s41562-021-01156-y 34211151

[R51] Feng C, & Liu C (2022). Resolving the limitations of the CNI model in moral decision making using the CAN algorithm: A methodological contrast. Behavioral Sciences, 12(7), 233. 10.3390/bs12070233 35877303 PMC9311620

[R52] Ferré P, Guasch M, Stadthagen-Gonzalez H, & Comesaña M (2022). Love me in L1, but hate me in L2: How native speakers and bilinguals rate the affectivity of words when feeling or thinking about them. Bilingualism: Language and Cognition, 25(5), 786–800. 10.1017/S1366728922000189

[R53] Fischer MH (2018). Why numbers are embodied concepts. Frontiers in Psychology, 8, 2347. 10.3389/fpsyg.2017.02347 29379459 PMC5775243

[R54] García AM, Moguilner S, Torquati K, García-Marco E, Herrera E, Muñoz E, Castillo EM, Kleineschay T, Sedeño L, & Ibáñez A (2019). How meaning unfolds in neural time: Embodied reactivations can precede multimodal semantic effects during language processing. NeuroImage, 197(May), 439–449. 10.1016/j.neuroimage.2019.05.002 31059796

[R55] García AM, de Leon J, Tee BL, Blasi DE, & Gorno-Tempini ML (2023). Speech and language markers of neurodegeneration: A call for global equity. Brain, 146(12), 4870–4879. 10.1093/brain/awad253 37497623 PMC10690018

[R56] Garcia O (2017). Bilingual education. In Coulmas F (Ed.), The handbook of sociolinguistics (pp. 405–420). Hoboken, NJ: John Wiley & Sons, Ltd. 10.1002/9781405166256.ch25

[R57] Garcia O, Faghihi N, Raola AR, & Vaid J (2021). Factors influencing bilinguals’ speed and accuracy of number judgments across languages: A meta-analytic review. Journal of Memory and Language, 118, 104211. 10.1016/j.jml.2020.104211

[R58] Gawronski B, Armstrong J, Conway P, Friesdorf R, & Hütter M (2017). Consequences, norms, and generalized inaction in moral dilemmas: The CNI model of moral decision-making. Journal of Personality and Social Psychology, 113(3), 343–376. 10.1037/pspa0000086 28816493

[R59] Gawronski B, Conway P, Hütter M, Luke DM, Armstrong J, & Friesdorf R (2020). On the validity of the CNI model of moral decision-making: Reply to Baron and Goodwin (2020). Judgment and Decision Making, 15(6), 1054–1072. 10.1017/S1930297500008251

[R60] Geipel J, Hadjichristidis C, & Surian L (2015). The foreign language effect on moral judgment: The role of emotions and norms. PLoS ONE, 10(7), 1–17. 10.1371/journal.pone.0131529 PMC4503530 26177508

[R61] Gollan TH, Weissbergr GH, Runnqvist E, Montoya RI, & Cera CM (2012). Self-ratings of spoken language dominance: A multilingual naming test (MINT) and preliminary norms for young and aging Spanish–English bilinguals. Bilingualism: Language and Cognition, 15(3), 594–615. 10.1017/S1366728911000332 25364296 PMC4212892

[R62] Goral M, Campanelli L, & Spiro A (2015). Language dominance and inhibition abilities in bilingual older adults. Bilingualism: Language and Cognition, 18(1), 79–89. 10.1017/S1366728913000126 27531968 PMC4983451

[R63] Grant A, Legault J, & Li P (2019). What do bilingual models tell us about the neurocognition of multiple languages?. In Schwieter JW, & Paradis M (Eds), The Handbook of the Neuroscience of Multilingualism. Hoboken, NJ: John Wiley & Sons, Ltd. (pp. 48–74). 10.1002/9781119387725.ch3

[R64] Greene JD (2014). The cognitive neuroscience of moral judgment and decision making. In Gazzaniga MS, & Mangun GR (Eds), The cognitive neurosciences (5th ed., pp. 1013–1023). Cambridge, MA: MIT Press. 10.7551/mitpress/9504.003.0110

[R65] Greene JD (2015). The rise of moral cognition. Cognition, 135, 39–42. 10.1016/j.cognition.2014.11.018 25498900

[R66] Greene JD, Sommerville RB, Nystrom LE, Darley JM, & Cohen JD (2001). An fMRI investigation of emotional engagement in moral judgment. Science, 293(5537), 2105–2108. 10.1126/science.1062872 11557895

[R67] Greene JD, Morelli SA, Lowenberg K, Nystrom LE, & Cohen JD (2008). Cognitive load selectively interferes with utilitarian moral judgment. Cognition, 107(3), 1144–1154. 10.1016/j.cognition.2007.11.004 18158145 PMC2429958

[R68] Gullifer JW, & Titone D (2020). Characterizing the social diversity of bilingualism using language entropy. Bilingualism: Language and Cognition, 23(2), 283–294. 10.1017/S1366728919000026

[R69] Gullifer JW, Kousaie S, Gilbert AC, Grant A, Giroud N, Coulter K, Klein D, Baum S, Phillips N, & Titone D (2021). Bilingual language experience as a multidimensional spectrum: Associations with objective and subjective language proficiency. Applied Psycholinguistics, 42(2), 245–278. 10.1017/S0142716420000521

[R70] Hadjichristidis C, Geipel J, & Keysar B (2019). The influence of native language in shaping judgment and choice. In Srinivasan N (Ed.), Progress in brain research (Vol. 247, pp. 253–272). Amsterdam, The Netherlands: Elsevier B.V. 10.1016/bs.pbr.2019.02.003 31196437

[R71] Harris CL, Gleason JB, & Ayçiçeǧi A (2006). 10. When is a first language more emotional? Psychophysiological evidence from bilingual speakers. In Pavlenko A (Ed.), Bilingual minds (Issue 1, pp. 257–283). Bristol, UK: Multilingual Matters. 10.21832/9781853598746-012

[R72] Hayakawa S, & Keysar B (2018). Using a foreign language reduces mental imagery. Cognition, 173, 8–15. 10.1016/j.cognition.2017.12.010 29278805

[R73] Hayakawa S, Costa A, Foucart A, & Keysar B (2016). Using a foreign language changes our choices. Trends in Cognitive Sciences, 20(11), 791–793. 10.1016/j.tics.2016.08.004 27600315

[R74] Hayakawa S, Tannenbaum D, Costa A, Corey JD, & Keysar B (2017). Thinking more or feeling less? Explaining the foreign-language effect on moral judgment. Psychological Science, 28(10), 1387–1397. 10.1177/0956797617720944 28806137

[R75] Hennig M, & Hütter M (2021). Consequences, norms, or willingness to interfere: A proCNI model analysis of the foreign language effect in moral dilemma judgment. Journal of Experimental Social Psychology, 95(April), 104148. 10.1016/j.jesp.2021.104148

[R76] Hoshino N, Dussias PE, & Kroll JF (2010). Processing subject-verb agreement in a second language depends on proficiency. Bilingualism: Language and Cognition, 13(2), 87–98. 10.1017/S1366728909990034 20640178 PMC2904637

[R77] Hui NY, Yuan M, Fong MCM, & Wang WSY (2020). L2 proficiency predicts inhibitory ability in L1-dominant speakers. International Journal of Bilingualism, 24(5–6), 984–998. 10.1177/1367006920914399

[R78] Hulstijn JH (2011). Language proficiency in native and nonnative speakers: An agenda for research and suggestions for second-language assessment. Language Assessment Quarterly, 8(3), 229–249. 10.1080/15434303.2011.565844

[R79] Hulstijn JH (2012). The construct of language proficiency in the study of bilingualism from a cognitive perspective. Bilingualism: Language and Cognition, 15(2), 422–423. 10.1017/S1366728911000678

[R80] Hulstijn JH (2020). Proximate and ultimate explanations of individual differences in language use and language acquisition. Dutch Journal of Applied Linguistics, 9(1–2), 21–37. 10.1075/dujal.19027.hul

[R81] Ibáñez A, Manes F, Escobar J, Trujillo N, Andreucci P, & Hurtado E (2010). Gesture influences the processing of figurative language in non-native speakers: ERP evidence. Neuroscience Letters, 471(1), 48–52. 10.1016/J.NEULET.2010.01.009 20079804

[R82] Imbault C, Titone D, Warriner AB, & Kuperman V (2021). How are words felt in a second language: Norms for 2,628 English words for valence and arousal by L2 speakers. Bilingualism: Language and Cognition, 24(2), 281–292. 10.1017/S1366728920000474

[R83] Inzlicht M, Bartholow BD, & Hirsh JB (2015). Emotional foundations of cognitive control. Trends in Cognitive Sciences, 19(3), 126–132. 10.1016/j.tics.2015.01.004 25659515 PMC4348332

[R84] Jankowiak K, & Korpal P (2018). On modality effects in bilingual emotional language processing: Evidence from galvanic skin response. Journal of Psycholinguistic Research, 47(3), 663–677. 10.1007/s10936-017-9552-5 29285592 PMC5937920

[R85] Kahane G (2015). Sidetracked by trolleys: Why sacrificial moral dilemmas tell us little (or nothing) about utilitarian judgment. Social Neuroscience, 10(5), 551–560. 10.1080/17470919.2015.1023400 25791902 PMC4642180

[R86] Kahane G, Everett JAC, Earp BD, Caviola L, Faber NS, Crockett MJ, & Savulescu J (2018). Beyond sacrificial harm: A two-dimensional model of utilitarian psychology. Psychological Review, 125(2), 131–164. 10.1037/rev0000093 29265854 PMC5900580

[R87] Kapa LL, & Colombo J (2013). Attentional control in early and later bilingual children. Cognitive Development, 28(3), 233. 10.1016/J.COGDEV.2013.01.011 24910499 PMC4044912

[R88] Kaushanskaya M, Blumenfeld HK, & Marian V (2020). The language experience and proficiency questionnaire (LEAP-Q): Ten years later. Bilingualism: Language and Cognition, 23(5), 945–950. 10.1017/S1366728919000038 33628083 PMC7899192

[R89] Keating GD (2017). L2 proficiency matters in comparative L1/L2 processing research. Bilingualism: Language and Cognition, 20(4), 700–701. 10.1017/S1366728916000912

[R90] Keshmirian A, Deroy O, & Bahrami B (2022). Many heads are more utilitarian than one. Cognition, 220, 104965. 10.1016/j.cognition.2021.104965 34872034

[R91] Keysar B, Hayakawa SL, & An SG (2012). The foreign-language effect. Psychological Science, 23(6), 661–668. 10.1177/0956797611432178 22517192

[R92] Klenk M (2021). The influence of situational factors in sacrificial dilemmas on utilitarian moral judgments: A systematic review and meta-analysis. Review of Philosophy and Psychology 17, 593–625. 10.1007/s13164-021-00547-4

[R93] Kneer M, & Hannikainen IR (2022). Trolleys, triage and COVID-19: The role of psychological realism in sacrificial dilemmas. Cognition and Emotion, 36(1), 137–153. 10.1080/02699931.2021.1964940 34392813

[R94] Kogan B, Muñoz E, Ibáñez A, & García AM (2020). Too late to be grounded? Motor resonance for action words acquired after middle childhood. Brain and Cognition, 138, 105509. 10.1016/j.bandc.2019.105509 31855702

[R95] Kootstra GJ, Van Hell JG, & Dijkstra T (2012). Priming of code-switches in sentences: The role of lexical repetition, cognates, and language proficiency. Bilingualism: Language and Cognition, 15(4), 797–819. 10.1017/S136672891100068X

[R96] Körner A, & Deutsch R (2023). Deontology and utilitarianism in real life: A set of moral dilemmas based on historic events. Personality and Social Psychology Bulletin, 49(10), 1511–1528. 10.1177/01461672221103058 35751175 PMC10478346

[R97] Körner A, Joffe S, & Deutsch R (2019). When skeptical, stick with the norm: Low dilemma plausibility increases deontological moral judgments. Journal of Experimental Social Psychology, 84, 103834. 10.1016/j.jesp.2019.103834

[R98] Körner A, Deutsch R, & Gawronski B (2020). Using the CNI model to investigate individual differences in moral dilemma judgments. Personality and Social Psychology Bulletin, 46(9), 1392–1407. 10.1177/0146167220907203 32111135

[R99] Kroneisen M, & Heck DW (2020). Interindividual differences in the sensitivity for consequences, moral norms, and preferences for inaction: Relating basic personality traits to the CNI model. Personality and Social Psychology Bulletin, 46(7), 1013–1026. 10.1177/0146167219893994 31889471 PMC7278365

[R100] Kunnari A, Sundvall JRI, & Laakasuo M (2020). Challenges in process dissociation measures for moral cognition. Frontiers in Psychology, 11, 3195. 10.3389/FPSYG.2020.559934/BIBTEX PMC7759142 33362623

[R101] Langdon HW, Wiig EH, & Nielsen NP (2005). Dual-dimension naming speed and language-dominance ratings by bilingual Hispanic adults. Bilingual Research Journal, 29(2), 319–336. 10.1080/15235882.2005.10162838

[R102] Leibovich T, Katzin N, Harel M, & Henik A (2017). From “sense of number” to “sense of magnitude”: The role of continuous magnitudes in numerical cognition. Behavioral and Brain Sciences, 40, e164. 10.1017/S0140525X16000960 27530053

[R103] Li P, & Jeong H (2020). The social brain of language: Grounding second language learning in social interaction. npj Science of Learning, 5, 8. 10.1038/s41539-020-0068-7 32595983 PMC7305321

[R104] Liberto GMD, Nie J, Yeaton J, Khalighinejad B, Shamma SA, & Mesgarani N (2021). Neural representation of linguistic feature hierarchy reflects second-language proficiency. NeuroImage, 227, 117586. 10.1016/j.neuroimage.2020.117586 33346131 PMC8527895

[R105] Lim N (2016). Cultural differences in emotion: Differences in emotional arousal level between the East and the West. Integrative Medicine Research, 5(2), 105–109. 10.1016/j.imr.2016.03.004 28462104 PMC5381435

[R106] Liu C, Wang H, Timmer K, & Jiao L (2022). The foreign language effect on altruistic decision making: Insights from the framing effect. Bilingualism: Language and Cognition 25(5), 890–898. 10.1017/S136672892200012

[R107] Lopes da Cunha P, Fittipaldi S, González Campo C, Kauffman M, Rodríguez-Quiroga S, Yacovino DA, Ibáñez A, Birba A, & García AM (2023). Social concepts and the cerebellum: Behavioural and functional connectivity signatures in cerebellar ataxic patients. Philosophical Transactions of the Royal Society B: Biological Sciences, 378(1870), 20210364. 10.1098/rstb.2021.0364 PMC9791482 36571119

[R108] Lucifora C, Martino G, Curcuruto A, Salehinejad MA, & Vicario CM (2021). How self-control predicts moral decision making: An exploratory study on healthy participants. International Journal of Environmental Research and Public Health, 18(7), 3840. 10.3390/ijerph18073840 33917567 PMC8038791

[R109] Luke DM, & Gawronski B (2022). Temporal stability of moral dilemma judgments: A longitudinal analysis using the CNI model. Personality and Social Psychology Bulletin, 48(8), 1191–1203. 10.1177/01461672211035024 34338077

[R110] Marian V, & Hayakawa S (2021). Measuring bilingualism: The quest for a bilingualism quotient. Applied Psycholinguistics, 42(2), 527–548. 10.1017/S0142716420000533 34054162 PMC8158058

[R111] Marian V, Blumenfeld HK, & Kaushanskaya M (2007). The language experience and proficiency questionnaire (LEAP-Q): Assessing language profiles in bilinguals and multilinguals. Journal of Speech, Language, and Hearing Research, 50(4), 940–967. 10.1044/1092-4388(2007/067) 17675598

[R112] McDonald MM, Defever AM, & Navarrete CD (2017). Killing for the greater good: Action aversion and the emotional inhibition of harm in moral dilemmas. Evolution and Human Behavior, 38(6), 770–778. 10.1016/j.evolhumbehav.2017.06.001

[R113] McFarlane S, & Perez HC (2020). Some challenges for research on emotion and moral judgement: The moral foreign-language effect as a case study. Diametros, 17(64), 56–71. 10.33392/diam.1476

[R114] McLean S, Stewart J, & Batty AO (2020). Predicting L2 reading proficiency with modalities of vocabulary knowledge: A bootstrapping approach. Language Testing, 37(3), 389–411. 10.1177/0265532219898380

[R115] Michelini Y, Acuña I, Guzmán JI, & Godoy JC (2019). Latemo-e: A film database to elicit discrete emotions and evaluate emotional dimensions in Latin-Americans. Trends in Psychology, 27(2), 473–490. 10.9788/TP2019.2-13

[R116] Miller D, Solis-Barroso C, & Delgado R (2021). The foreign language effect in bilingualism: Examining prosocial sentiment after offense taking. Applied Psycholinguistics, 42(2), 395–416. 10.1017/S0142716420000806

[R117] Miozzo M, Navarrete E, Ongis M, Mello E, Girotto V, & Peressotti F (2020). Foreign language effect in decision-making: How foreign is it? Cognition, 199, 104245. 10.1016/j.cognition.2020.104245 32222524

[R118] Miri MA, & Pishghadam R (2021). Toward an emotioncy based education: A systematic review of the literature. Frontiers in Psychology, 12, 727186. 10.3389/fpsyg.2021.727186 34421775 PMC8374051

[R119] Nasello JA, & Triffaux J (2023). The role of empathy in trolley problems and variants: A systematic review and meta-analysis. British Journal of Social Psychology, 62(4), 1753–1781. 10.1111/bjso.12654 37314211

[R120] Navajas J, Heduan FÁ, Garbulsky G, Tagliazucchi E, Ariely D, & Sigman M (2021). Moral responses to the COVID-19 crisis. Royal Society Open Science, 8(9), 210096. 10.1098/rsos.210096 34527267 PMC8439416

[R121] Nguyen TK, & Astington JW (2014). Reassessing the bilingual advantage in theory of mind and its cognitive underpinnings. Bilingualism: Language and Cognition, 17(2), 396–409. 10.1017/S1366728913000394

[R122] Oh TM, Graham S, Ng P, Yeh IB, Chan BPL, & Edwards AM (2019). Age and proficiency in the bilingual brain revisited: Activation patterns across different L2-learner types. Frontiers in Communication 4, 39. 10.3389/FCOMM.2019.00039

[R123] Okon-Singer H, Hendler T, Pessoa L, & Shackman AJ (2015). The neurobiology of emotion-cognition interactions: Fundamental questions and strategies for future research. Frontiers in Human Neuroscience, 9, 58. 10.3389/fnhum.2015.00058 25774129 PMC4344113

[R124] Olson DJ (2023). A systematic review of proficiency assessment methods in bilingualism research. International Journal of Bilingualism 28(2), 163–187. 10.1177/13670069231153720

[R125] Page MJ, McKenzie JE, Bossuyt PM, Boutron I, Hoffmann TC, Mulrow CD, Shamseer L, Tetzlaff JM, Akl EA, Brennan SE, Chou R, Glanville J, Grimshaw JM, Hróbjartsson A, Lalu MM, Li T, Loder EW, Mayo-Wilson E, Mcdonald S, … Moher D (2021). The PRISMA 2020 statement: An updated guideline for reporting systematic reviews. The BMJ, 372, n71. 10.1136/bmj.n71 33782057 PMC8005924

[R126] Park HI, Solon M, Dehghan-Chaleshtori M, & Ghanbar H (2022). Proficiency reporting practices in research on second language acquisition: Have we made any progress? Language Learning, 72(1), 198–236. 10.1111/lang.12475

[R127] Pattij T, & Schoffelmeer ANM (2015). Serotonin and inhibitory response control: Focusing on the role of 5-HT1A receptors. European Journal of Pharmacology, 753, 140–145. 10.1016/j.ejphar.2014.05.064 25094037

[R128] Pavlenko A (2017). Do you wish to waive your rights? Affect and decision-making in multilingual speakers. Current Opinion in Psychology, 17, 74–78. 10.1016/j.copsyc.2017.06.005 28950977

[R129] Pearson J, Naselaris T, Holmes EA, & Kosslyn SM (2015). Mental imagery: Functional mechanisms and clinical applications. Trends in Cognitive Sciences, 19(10), 590–602. 10.1016/j.tics.2015.08.003 26412097 PMC4595480

[R130] Petersen IT, Hoyniak CP, McQuillan ME, Bates JE, & Staples AD (2016). Measuring the development of inhibitory control: The challenge of heterotypic continuity. Developmental Review, 40, 25–71. 10.1016/j.dr.2016.02.001 27346906 PMC4917209

[R131] Pfattheicher S, Nielsen YA, & Thielmann I (2022). Prosocial behavior and altruism: A review of concepts and definitions. Current Opinion in Psychology, 44, 124–129. 10.1016/j.copsyc.2021.08.021 34627110

[R132] Pishghadam R, Adamson B, & Shayesteh S (2013). Emotion-based language instruction (EBLI) as a new perspective in bilingual education. Multilingual Education, 3(1), 9. 10.1186/2191-5059-3-9

[R133] Reese M, Bryant D, & Ethridge L (2020). Biomarkers for moral cognition: Current status and future prospects for neurotransmitters and neuropeptides. In Neuroscience and biobehavioral reviews (Vol. 113, pp. 88–97). Elsevier Ltd. 10.1016/j.neubiorev.2020.03.009 32171842

[R134] Riva P, Manfrinati A, Sacchi S, Pisoni A, & Romero Lauro LJ (2019). Selective changes in moral judgment by noninvasive brain stimulation of the medial prefrontal cortex. Cognitive, Affective, & Behavioral Neuroscience, 19(4), 797–810. 10.3758/s13415-018-00664-1 30411201

[R135] Santilli M, Vilas MG, Mikulan E, Martorell Caro M, Muñoz E, Sedeño L, Ibáñez A, & García AM (2019). Bilingual memory, to the extreme: Lexical processing in simultaneous interpreters. Bilingualism: Language and Cognition, 22(2), 331–348. 10.1017/S1366728918000378

[R136] Sato M (2017). Interaction mindsets, interactional behaviors, and L2 development: An affective-social-cognitive model. Language Learning, 67(2), 249–283. 10.1111/lang.12214

[R137] Schaich Borg J, Hynes C, Van Horn J, Grafton S, & Sinnott-Armstrong W (2006). Consequences, action, and intention as factors in moral judgments: An fMRI investigation. Journal of Cognitive Neuroscience, 18(5), 803–817. 10.1162/jocn.2006.18.5.803 16768379

[R138] Schiller D, Yu ANC, Alia-Klein N, Becker S, Cromwell HC, Dolcos F, Eslinger PJ, Frewen P, Kemp AH, Pace-Schott EF, Raber J, Silton RL, Stefanova E, Williams JHG, Abe N, Aghajani M, Albrecht F, Alexander R, Anders S, … Leonie L (2023). The human affectome. Neuroscience & Biobehavioral Reviews 158, 105450. 10.1016/j.neubiorev.2023.105450 37925091 PMC11003721

[R139] Shin HI, & Kim J (2017). Foreign language effect and psychological distance. Journal of Psycholinguistic Research, 46(6), 1339–1352. 10.1007/s10936-017-9498-7 28516209

[R140] Stankovic M, Biedermann B, & Hamamura T (2022). Not all bilinguals are the same: A meta-analysis of the moral foreign language effect. Brain and Language, 227, 105082. 10.1016/j.bandl.2022.105082 35093765

[R141] Sulpizio S, Del Maschio N, Del Mauro G, Fedeli D, & Abutalebi J (2020). Bilingualism as a gradient measure modulates functional connectivity of language and control networks. NeuroImage, 205, 116306. 10.1016/j.neuroimage.2019.116306 31654763

[R142] Sutton TM, Altarriba J, Gianico JL, & Basnight-Brown DM (2007). The automatic access of emotion: Emotional Stroop effects in Spanish–English bilingual speakers. Cognition and Emotion, 21(5), 1077–1090. 10.1080/02699930601054133

[R143] Takamatsu R (2018). Turning off the empathy switch: Lower empathic concern for the victim leads to utilitarian choices of action. PLoS ONE, 13(9), e0203826. 10.1371/journal.pone.0203826 30212541 PMC6136766

[R144] Tasso A, Sarlo M, & Lotto L (2017). Emotions associated with counterfactual comparisons drive decision-making in footbridge-type moral dilemmas. Motivation and Emotion, 41(3), 410–418. 10.1007/s11031-017-9607-9

[R145] Tassy S, Oullier O, Mancini J, & Wicker B (2013). Discrepancies between judgment and choice of action in moral dilemmas. Frontiers in Psychology, 4, 250. 10.3389/fpsyg.2013.00250 23720645 PMC3655270

[R146] Thanissery N, Parihar P, & Kar BR (2020). Language proficiency, sociolinguistic factors and inhibitory control among bilinguals. Journal of Cultural Cognitive Science, 4(2), 217–241. 10.1007/s41809-020-00065-2

[R147] Thomson JJ (1976). Killing, letting die, and the trolley problem. Monist, 59(2), 204–217. 10.5840/monist197659224 11662247

[R148] Thomson JJ (1985). The trolley problem. The Yale Law Journal, 94(6), 1395. 10.2307/796133

[R149] Titone DA, & Tiv M (2022). Rethinking multilingual experience through a systems framework of bilingualism. Bilingualism: Language and Cognition. Advance online publication 1–16. 10.1017/S1366728921001127

[R150] Tomoschuk B, Ferreira VS, & Gollan TH (2019). When a seven is not a seven: Self-ratings of bilingual language proficiency differ between and within language populations. Bilingualism: Language and Cognition, 22(3), 516–536. 10.1017/S1366728918000421

[R151] Tversky A, & Kahneman D (1981). The framing of decisions and the psychology of choice. Science, 211(4481), 453–458. 10.1126/science.7455683 7455683

[R152] Van Bavel JJ, FeldmanHall O, & Mende-Siedlecki P (2015). The neuroscience of moral cognition: From dual processes to dynamic systems. In Current opinion in psychology (Vol. 6, pp. 167–172). Elsevier B.V. 10.1016/j.copsyc.2015.08.009

[R153] van den Bos K, Müller PA, & Damen T (2011). A behavioral disinhibition hypothesis of interventions in moral dilemmas. Emotion Review, 3(3), 281–283. 10.1177/1754073911402369

[R154] Van Rinsveld A, Schiltz C, Landerl K, Brunner M, & Ugen S (2016). Speaking two languages with different number naming systems: What implications for magnitude judgments in bilinguals at different stages of language acquisition? Cognitive Processing, 17(3), 225–241. 10.1007/s10339-016-0762-9 27020298

[R155] Veríssimo J (2021). Analysis of rating scales: A pervasive problem in bilingualism research and a solution with Bayesian ordinal models. Bilingualism: Language and Cognition, 24(5), 842–848. 10.1017/S1366728921000316

[R156] Vukovic N (2013). When words get physical: Evidence for proficiency-modulated somatotopic motor interference during second language comprehension. Proceedings of the Annual Meeting of the Cognitive Science Society, 35. Retrieved from https://escholarship.org/uc/item/0jb6s58t

[R157] Wagner E (2013). Assessing listening. In Kunnan AJ (Ed.), The companion to language assessment (pp. 47–63). Hoboken, NJ: John Wiley & Sons, Ltd. 10.1002/9781118411360.wbcla094

[R158] Wartenburger I, Heekeren HR, Abutalebi J, Cappa SF, Villringer A, & Perani D (2003). Early setting of grammatical processing in the bilingual brain. Neuron, 37(1), 159–170. 10.1016/S0896-6273(02)01150-9 12526781

[R159] Winskel H, & Bhatt D (2020). The role of culture and language in moral decision-making. Culture and Brain, 8(2), 207–225. 10.1007/s40167-019-00085-y

[R160] Wong D (2019). Definition of morality. In LaFollette H (Ed.), International encyclopedia of ethics (pp. 1–9). Hoboken, NJ: John Wiley & Sons, Ltd. 10.1002/9781444367072.wbiee671.pub2

[R161] Wong G, & Ng BC (2018). Moral judgement in early bilinguals: Language dominance influences responses to moral dilemmas. Frontiers in Psychology, 9, 1070. 10.3389/fpsyg.2018.01070 30002639 PMC6032433

[R162] Xue SW, Wang Y, & Tang YY (2013). Personal and impersonal stimuli differentially engage brain networks during moral reasoning. Brain and Cognition, 81(1), 24–28. 10.1016/j.bandc.2012.09.004 23164731

[R163] Yik M, Mues C, Sze INL, Kuppens P, Tuerlinckx F, De Roover K, Kwok FHC, Schwartz SH, Abu-Hilal M, Adebayo DF, Aguilar P, Al-Bahrani M, Anderson MH, Andrade L, Bratko D, Bushina E, Choi JW, Cieciuch J, Dru V, … Russell JA (2023). On the relationship between valence and arousal in samples across the globe. Emotion, 23(2), 332–344. 10.1037/emo0001095 35446055

[R164] Youssef FF, Dookeeram K, Basdeo V, Francis E, Doman M, Mamed D, Maloo S, Degannes J, Dobo L, Ditshotlo P, & Legall G (2012). Stress alters personal moral decision making. Psychoneuroendocrinology, 37(4), 491–498. 10.1016/j.psyneuen.2011.07.017 21899956

[R165] Yu H, Siegel JZ, & Crockett MJ (2019). Modeling morality in 3-D: Decision-making, judgment, and inference. Topics in Cognitive Science, 11(2), 409–432. 10.1111/tops.12382 31042018 PMC6519237

[R166] Zell E, & Krizan Z (2014). Do people have insight into their abilities? A metasynthesis. Perspectives on Psychological Science, 9(2), 111–125. 10.1177/1745691613518075 26173249

[R167] Zeybek T (2021). An investigation into the foreign language effect in decision making and judgment [Master’s thesis]. Pamukkale University, Denizli, Turkey.

[R168] Zhang L, Kong M, Li Z, Zhao X, & Gao L (2018). Chronic stress and moral decision-making: An exploration with the CNI model. Frontiers in Psychology, 9, 1702. 10.3389/fpsyg.2018.01702 30254597 PMC6141736

[R169] Zheng L, Mobbs D, & Yu R (2020). The behavioral and neural basis of foreign language effect on risk-taking. Neuropsychologia, 136(November 2019), 107290. 10.1016/j.neuropsychologia.2019.107290 31794712

