# Supplementary material for: Bilinguals on the footbridge: the role of foreign-language proficiency in moral decision making
Source: Biling (Camb Engl). Author manuscript; Available in PMC 2025 Oct 25. (PMC11133874; doi:10.1017/S1366728924000312)
Supplement: Supplementary Material [file NIHMS1998256-supplement-Supplementary_Material.pdf]

Supplementary material

1. Summary of papers in the corpus

Table 1. Summary of papers with evidence on the role of second-language proficiency on the foreign moral language effect.

Study	Sample description	Stimuli and conditions	Type of task	Results regarding moral FLE and proficiency	Other relevant results and procedures
Costa et al. (2014), Exp. 1	112 English/Spanish, 80 Korean/English, and 18 Spanish or English/Hebrew bilinguals (age: 21; L2p: 52.5% [reading])	Footbridge dilemma (L1 or L2).	Written presentation, with pictures. Answering whether they would push the man (Yes or No).	More odds of utilitarian choices on L2 than L1.	
Costa et al. (2014), Exp. 2, impersonal	397 Spanish/English bilinguals (age: 21; L2p: 71.67% [reading])	Switch/trolley dilemma (L1 or L2).	Written presentation. Answering whether they would press the switch (Yes or No).	No significant differences between languages.	
Costa et al. (2014), Exp. 2, personal	397 Spanish/English bilinguals (age: 21; L2p: 71.67% [reading])	Footbridge dilemma (L1 or L2).	Written presentation. Answering whether they would push the man (Yes or No).	More odds of utilitarian choices on L2 than L1. Divided between lower and higher L2p groups, the lower L2p group showed more odds of utilitarian choices than the high L2p group.	
Costa et al. (2014), Exp. 2, impersonal	328 English/Spanish bilinguals (age: 21; L2p: 65% [reading])	Switch/trolley dilemma (L1 or L2).	Written presentation. Answering whether they would press the switch (Yes or No).	No significant differences between languages.	

Costa et al. (2014), Exp. 2, personal	328 English/Spanish bilinguals (age: 21; L2p: 65% [reading])	Footbridge dilemma (L1 or L2).	Written presentation. Answering whether they would push the man (Yes or No).	More odds of utilitarian choices on L2 than L1. Divided between lower and higher L2p groups, the lower L2p group showed more odds of utilitarian choices than the high L2p group.	
Geipel et al. (2015), Exp. 1, impersonal	76 Italian/English bilinguals (Overall age: 22.08; L2p: 58.25% [overall])	Switch/trolley dilemma (L1 or L2).	Written presentation. Answering whether they should press the switch (Yes or No).	No significant differences between languages.	Used CEFR for an objective L2p cut-off
Geipel et al. (2015), Exp. 1, personal	76 Italian/English bilinguals (Overall age: 22.08; L2p: 58.25% [overall])	Footbridge dilemma (L1 or L2).	Written presentation. Answering whether they should push the man (Yes or No).	More odds of utilitarian choices on L2 than L1. Lower L2p correlated negatively with odds of making utilitarian choices.	Used CEFR for an objective L2p cut-off
Geipel et al. (2015), Exp. 1, impersonal	68 Italian/German bilinguals (Overall age: 22.08; L2p: 69.75% [overall]) (overall age: 22.08)	Switch/trolley dilemma (L1 or L2).	Written presentation. Answering whether they should press the switch (Yes or No).	No significant differences between languages.	Used CEFR for an objective L2p cut-off
Geipel et al. (2015), Exp. 1, personal	68 Italian/German bilinguals (Overall age: 22.08; L2p: 69.75% [overall]) (overall age: 22.08)	Footbridge dilemma (L1 or L2).	Written presentation. Answering whether they should push the man (Yes or No).	More odds of utilitarian choices on L2 than L1. Lower L2p correlated negatively with odds of making utilitarian choices.	Used CEFR for an objective L2p cut-off
Geipel et al. (2015), Exp. 2, impersonal	161 Chinese/English bilinguals (age: 23.41; L2p: 53.75% [overall])	Switch/trolley dilemma (L1 or L2).	Written presentation. Answering whether they should press the switch (Yes or No). Ranking three distress scales (1-7). Ranking the appropiateness of pressing the switch (1-7)	No significant differences between languages.	Used CET-4 for an objective L2p cut-off. Distress ratings were lower on L2. Distress ratings were higher for switch than footbridge. No judgement differences between languages.

Geipel et al. (2015), Exp. 2, personal	161 Chinese/English bilinguals (age: 23.41; L2p: 53.75% [overall])	Footbridge dilemma (L1 or L2).	Written presentation. Answering whether they should push the man (Yes or No). Ranking three distress scales (1-7). Ranking the appropiateness of pushing the man (1-7)	More odds of utilitarian choices on L2 than L1. Lower L2p correlated negatively with odds of making utilitarian choices.	Used CET-4 for an objective L2p cut-off. Distress ratings were lower on L2. Distress ratings were higher for switch than footbridge. Higher utilitarianism regarding judgement on L2 than L1.
Chan et al. (2016)	144 Chinese/English bilinguals (age: 20.5; L2p: 68.3% [reading])	39 moral dilemmas (22 personal and 17 impersonal, on L1 or L2).	Written presentation of each dilemma randomly. Answering what would they do (Utilitarian or Deontological choice). Ranking arousal (1-9), valence 1-9), and the vividness of the image related to the dilemma (1-7)	No significant differences between languages.	More arousal and vividness of image on L2 than L1.
Chan et al. (2016), personal	144 Chinese/English bilinguals (age: 20.5; L2p: 68.3% [reading])	22 personal moral dilemmas (L1 or L2).	Written presentation of each dilemma randomly. Answering what would they do (Utilitarian or Deontological choice). Ranking arousal (1-9), valence 1-9), and the vividness of the image related to the dilemma (1-7)	No significant differences between languages.	
Chan et al. (2016), footbridge	144 Chinese/English bilinguals (age: 20.5; L2p: 68.3% [reading])	Footbridge dilemma (L1 or L2).	Written presentation. Answering what would they do (Utilitarian or Deontological choice). Ranking arousal (1-9), valence 1-9), and the vividness of the image related to the dilemma (1-7)	More odds of utilitarian choices on L2 than L1.	

Shin and Kim (2017), Exp. 1	161 Korean/English bilinguals (age: 20.2; L2p: 55.33% [reading])	Switch/trolley dilemma (L1 or L2).	Written presentation. Answering whether they should take action (Yes or No).	No significant differences between languages.
Shin and Kim (2017), Exp. 1	161 Korean/English bilinguals (age: 20.2; L2p: 55.33% [reading])	Fumes dilemma (L1 or L2).	Written presentation. Answering whether they should take action (Yes or No).	No significant differences between languages.
Shin and Kim (2017), Exp. 1	161 Korean/English bilinguals (age: 20.2; L2p: 55.33% [reading])	Baby dilemma (L1 or L2).	Written presentation. Answering whether they should take action (Yes or No).	More odds of utilitarian choices on L2 than L1. Lower L2p correlated negatively with odds of making utilitarian choices.
Shin and Kim (2017), Exp. 1	161 Korean/English bilinguals (age: 20.2; L2p: 55.33% [reading])	Transplant dilemma (L1 or L2).	Written presentation. Answering whether they should take action (Yes or No).	More odds of utilitarian choices on L2 than L1. Lower L2p correlated negatively with odds of making utilitarian choices.
Brouwer (2019), Exp. 1, impersonal	60 Dutch/English bilinguals (age: 27.8; L2p: 70% [reading])	Lost Wallet, Switch, and Taxes dilemmas (L1 or L2).	Written presentation. Answering whether they should take action (Yes or No).	No significant differences between languages.
Brouwer (2019), Exp. 1, personal	60 Dutch/English bilinguals (age: 27.8; L2p: 70% [reading])	Crying Baby, Footbridge, and Vitamins dilemmas (L1 or L2).	Written presentation. Answering whether they should take action (Yes or No).	No significant differences between languages.
Brouwer (2019), Exp. 2, impersonal	60 Dutch/English bilinguals (age: 29.5; L2p: 66.75% [listening])	Lost Wallet, Switch, and Taxes dilemmas (L1 or L2).	Auditory presentation. Answering whether they should take action (Yes or No).	More odds of utilitarian choices on L2 than L1.

Brouwer (2019), Exp. 2, personal	60 Dutch/English bilinguals (age: 29.5; L2p: 66.75% [listening])	Crying Baby, Footbridge, and Vitamins dilemmas (L1 or L2).	Auditory presentation. Answering whether they should take action (Yes or No).	More odds of utilitarian choices on L2 than L1.
Dylman and Champoux-Larsson (2020), Exp. 2a	198 Swedish/English bilinguals (age: 32.8; L2p: 77.77% [reading])	Footbridge dilemma (L1 or L2).	Written presentation. Answering whether they would push the man (Yes or No).	No significant differences between languages.
Dylman and Champoux-Larsson (2020), Exp. 2b	175 Swedish/French bilinguals (age: 35.2; L2p: 48.88% [reading])	Footbridge dilemma (L1 or L2).	Written presentation. Answering whether they would push the man (Yes or No).	More odds of utilitarian choices on L2 than L1.
Dylman and Champoux-Larsson (2020), Exp. 3a	305 Swedish/Norwegian bilinguals (age: 33.7; L2p: 62.22% [reading])	Footbridge dilemma (L1 or L2).	Written presentation. Answering whether they would push the man (Yes or No).	No significant differences between languages.
Dylman and Champoux-Larsson (2020), Exp. 3b	295 Norwegian/Swedish bilinguals (age: 33.6; L2p: 70% [reading])	Footbridge dilemma (L1 or L2).	Written presentation. Answering whether they would push the man (Yes or No).	No significant differences between languages.

Hayakawa et al. (2017), Exp. 1	214 German/English bilinguals (age: not reported; L2p: 66% [overall])	10 congruent and 10 incongruent moral dilemmas (L1 or L2).	Written presentation. Answering if it would be appropiate for them to push the man (Yes or No).	No significant differences between languages for traditional utilitarianism ("yes" responses to incongruent dilemmas). Blunted deontology effect on L2 for a process-dissociation model. No significant proficiency effect.
Hayakawa et al. (2017), Exp. 2	242 English/Spanish bilinguals (age: not reported; L2p: 73,17% [overall])	10 congruent and 10 incongruent moral dilemmas (L1 or L2).	Written presentation. Answering if it would be appropiate for them to push the man (Yes or No).	No significant differences between languages for traditional utilitarianism ("yes" responses to incongruent dilemmas). Blunted deontology and heightened utilitarianism effects on L2 for a process-dissociation model. Mildly significant inverse correlation on proficiency and utilitarianism.
Hayakawa et al. (2017), Exp. 3	195 Spanish/English bilinguals (age: not reported; L2p: 75.5% [overall])	10 congruent and 10 incongruent moral dilemmas (L1 or L2).	Written presentation. Answering if it would be appropiate for them to push the man, with reaffirmation of all consequences (Yes or No).	No significant differences between languages for traditional utilitarianism ("yes" responses to incongruent dilemmas). Heightened utilitarianism and nonsignificant (but robust across experiments) blunted deontology effect on L2 for a process-dissociation model. No significant proficiency effect.
Hayakawa et al. (2017), Exp. 4	211 German/English bilinguals (age: not reported; L2p: 65.83% [overall])	10 congruent and 10 incongruent moral dilemmas (L1 or L2).	Written presentation. Answering if it would be appropiate for them to push the man, with reaffirmation of all consequences (Yes or No).	No significant differences between languages for traditional utilitarianism ("yes" responses to incongruent dilemmas). Nonsignificant (but robust across experiments) blunted deontology effect on L2 for a process-dissociation model. No significant proficiency effect.

Hayakawa et al. (2017), Exp. 5	209 German/English bilinguals (age: not reported; L2p: 69.83% [overall])	10 congruent and 10 incongruent moral dilemmas (L1 or L2).	Written presentation. Answering whether they would push the man (Yes or No).	No significant differences between languages for traditional utilitarianism (“yes” responses to incongruent dilemmas). Blunted deontology effect on L2 for a process-dissociation model. No significant proficiency effect.	
Hayakawa et al. (2017), Exp. 6	206 English/German bilinguals (age: not reported; L2p: 69.5% [overall])	10 congruent and 10 incongruent moral dilemmas (L1 or L2).	Written presentation. Answering whether they would push the man (Yes or No).	No significant differences between languages for traditional utilitarianism (“yes” responses to incongruent dilemmas). Blunted deontology and heightened utilitarianism effects on L2 for a process-dissociation model. Significant inverse correlation on proficiency and utilitarianism.	
Corey et al. (2017), Exp. 1a, impersonal	211 Spanish/English bilinguals (age: not reported; L2p: 73.33% [reading])	Switch/trolley dilemma (L1 or L2). Vocabulary knowledge test.	Written presentation. Answering whether they would press the switch (Yes or No). Translating 10 dilemma relevant words to English.	More odds of utilitarian choices on L2 than L1. Divided between lower and higher L2p groups, the lower L2p group showed more odds of utilitarian choices than the L1 group, only for the vocabulary results, self-reported L2p yielded non-significant differences.	Evaluated L2p also with a vocabulary test
Corey et al. (2017), Exp. 1a, personal	211 Spanish/English bilinguals (age: not reported; L2p: 73.33% [reading])	Footbridge dilemma (L1 or L2). Vocabulary knowledge test.	Written presentation. Answering whether they would push the man (Yes or No). Translating 10 dilemma relevant words to English.	More odds of utilitarian choices on L2 than L1. Divided between lower and higher L2p groups, the lower L2p group showed more odds of utilitarian choices than the high L2p and L1 groups, only for the vocabulary results, self-reported L2p yielded non-significant differences.	Evaluated L2p also with a vocabulary test

Corey et al. (2017), Exp. 1b, impersonal	173 Spanish/English bilinguals (age: not reported; L2p: 70% [reading])	Hospital dilemma (L1 or L2).	Written presentation. Answering whether they would kill the one patient (Yes or No).	No significant differences between languages.	
Corey et al. (2017), Exp. 1b, personal	173 Spanish/English bilinguals (age: not reported; L2p: 70% [reading])	Terrorist dilemma (L1 or L2).	Written presentation. Answering whether they would kill the tourist (Yes or No).	More odds of utilitarian choices on L2 than L1.	
Corey et al. (2017), Exp. 2a, impersonal	204 Spanish/English bilinguals (age: not reported; L2p: 70% [reading])	Switch/trolley dilemma (L1 or L2, footbridge presented on the other language).	Written presentation. Answering whether they would press the switch (Yes or No).	No significant differences between languages. Divided between lower and higher L2p groups, the lower L2p group showed more odds of utilitarian choices than the high L2p group.	No language switching effect for decisions.
Corey et al. (2017), Exp. 2a, personal	204 Spanish/English bilinguals (age: not reported; L2p: 70% [reading])	Footbridge dilemma (L1 or L2, switch presented on the other language).	Written presentation. Answering whether they would push the man (Yes or No).	More odds of utilitarian choices on L2 than L1.	No language switching effect for decisions.
Corey et al. (2017), Exp. 2b, impersonal	399 Spanish/English bilinguals (age: not reported; L2p: 75% [reading])	Modified switch/trolley dilemma (L1 or L2, and Spanish or American nationalities for the 5 victims).	Written presentation. Answering whether they would press the switch (Yes or No).	No significant differences between languages.	No effect of the nationalities of the victims for decisions.

Corey et al. (2017), Exp. 2b, personal	399 Spanish/English bilinguals (age: not reported; L2p: 75% [reading])	Modified footbridge dilemma (L1 or L2, and Spanish or American nationalities for the 5 victims).	Written presentation. Answering whether they would push the man (Yes or No).	More odds of utilitarian choices on L2 than L1.	No effect of the nationalities of the victims for decisions.
Corey et al. (2017), Exp. 3a, impersonal	202 Spanish/English bilinguals (age: not reported; L2p: 65% [reading])	Switch/trolley dilemma (L1 or L2).	Written presentation. Answering whether they would press the switch (Yes or No).	No significant differences between languages. Divided between lower and higher L2p groups, the lower L2p group showed more odds of utilitarian choices than the high L2p group.	
Corey et al. (2017), Exp. 3a, personal	202 Spanish/English bilinguals (age: not reported; L2p: 65% [reading])	Modified footbridge dilemma (L1 or L2): "Button" dilemma where you do not push the man, pressing the button makes him fall into the rails.	Written presentation. Answering if they would press the button (Yes or No).	More odds of utilitarian choices on L2 than L1. Raw lower difference between L2 and L1 than on regular footbdridge (Exp. 1a)	
Corey et al. (2017), Exp. 3b, impersonal	190 Spanish/English bilinguals (age: not reported; L2p: 68.33% [reading])	Modified switch/trolley dilemma (L1 or L2): question changed to "Would you let five people die?"	Written presentation. Answering whether they would let five people die (Yes or No).	No significant differences between languages.	
Corey et al. (2017), Exp. 3b, personal	190 Spanish/English bilinguals (age: not reported; L2p: 68.33% [reading])	Modified footbridge dilemma (L1 or L2): question changed to "Would you let five people die?"	Written presentation. Answering whether they would let five people die (Yes or No).	More odds of utilitarian choices on L2 than L1.	

Corey et al. (2017), Exp. 3c, impersonal	201 Spanish/English bilinguals (age: not reported; L2p: 76.66% [reading])	Modified switch/trolley dilemma (L1 or L2): question changed to “Would you let five people die by not changing the track?"	Written presentation. Answering whether they would press the switch (Yes or No).	No significant differences between languages.
Corey et al. (2017), Exp. 3c, personal	201 Spanish/English bilinguals (age: not reported; L2p: 76.66% [reading])	Modified footbridge dilemma (L1 or L2): question changed to “Would you let five people die by not pushing him?”	Written presentation. Answering whether they would push the man (Yes or No).	No significant differences between languages. Divided between lower and higher L2p groups, the lower L2p group showed more odds of utilitarian choices than the high L2p group. The high L2p group failed to show FLE.
Corey et al. (2017), Exp. 3d, impersonal	197 Spanish/English bilinguals (age: not reported; L2p: 76.66% [reading])	Switch/trolley dilemma (L1 or L2).	Written presentation. Answering whether they would press the switch (Yes or No).	No significant differences between languages.
Corey et al. (2017), Exp. 3d, personal	197 Spanish/English bilinguals (age: not reported; L2p: 76.66% [reading])	Modified footbridge dilemma (L1 or L2): if pushed, the man will not die, instead, he will be disabled for life.	Written presentation. Answering whether they would push the man (Yes or No).	More odds of utilitarian choices on L2 than L1.

Białek et al. (2019)	204 Polish/English bilinguals, 138 Polish/German bilinguals, 163 Polish/Spanish bilinguals, 129 Polish/French bilinguals (age: 21.75; L2p: 70.33% [overall])	24 moral dilemmas composed of 6 dilemmas with 4 variations on proscriptive/prescriptive norms and action benefits over/under overall well-being	Written presentation. Answering whether they would perform the action described on each dilemma (Yes or No).	No significant differences between languages for traditional analysis (moral dilemmas involving a proscriptive norm that prohibits action in cases where the benefits of action outweigh its costs to well-being). Blunted deontology and heightened utilitarianism effects on L2 for a process-dissociation model. Sensitivity to consequences and norms were significantly lower on L2 for a CNI model.	
Brouwer (2021), impersonal	154 Dutch/English bilinguals (age: 24.1; L2p: 75.5% [overall])	Lost Wallet, Switch, and Taxes dilemmas (L1 or L2).	Written and auditory presentation. Answering whether they should take action (Yes or No).	No significant differences between languages.	More odds of utilitarian choices on auditory modality than written modality
Brouwer (2021), personal	154 Dutch/English bilinguals (age: 24.1; L2p: 75.5% [overall])	Crying Baby, Footbridge, and Vitamins dilemmas (L1 or L2).	Written and auditory presentation. Answering whether they should take action (Yes or No).	More odds of utilitarian choices on L2 than L1.	More odds of utilitarian choices on auditory modality than written modality
Winskel and Bhatt (2020), impersonal	166 Hindi/English bilinguals (age: 33.4; L2p: 91.43% [reading])	Switch/trolley dilemma (L1 or L2).	Written presentation. Answering whether they would pull the lever (Yes or No).	No significant differences between languages.	
Winskel and Bhatt (2020), impersonal	166 Hindi/English bilinguals (age: 33.4; L2p: 91.43% [reading])	Impersonal water park show dilemma (L1 or L2)	Written presentation. Answering whether they would press the switch (Yes or No).	No significant differences between languages.	

Winskel and Bhatt (2020), impersonal	166 Hindi/English bilinguals (age: 33.4; L2p: 91.43% [reading])	Impersonal family game dilemma (L1 or L2)	Written presentation. Answering whether they would press the switch (Yes or No).	No significant differences between languages.
Winskel and Bhatt (2020), personal	166 Hindi/English bilinguals (age: 33.4; L2p: 91.43% [reading])	Footbridge dilemma (L1 or L2)	Written presentation. Answering whether they would push the man (Yes or No).	No significant differences between languages.
Winskel and Bhatt (2020), personal	166 Hindi/English bilinguals (age: 33.4; L2p: 91.43% [reading])	Personal water park dilemma (L1 or L2)	Written presentation. Answering whether they would push the man (Yes or No).	No significant differences between languages.
Winskel and Bhatt (2020), personal	166 Hindi/English bilinguals (age: 33.4; L2p: 91.43% [reading])	Personal family game dilemma (L1 or L2)	Written presentation. Answering whether they would push the man (Yes or No).	No significant differences between languages.

2. Overview of the moral foreign language effect across all studies in the corpus

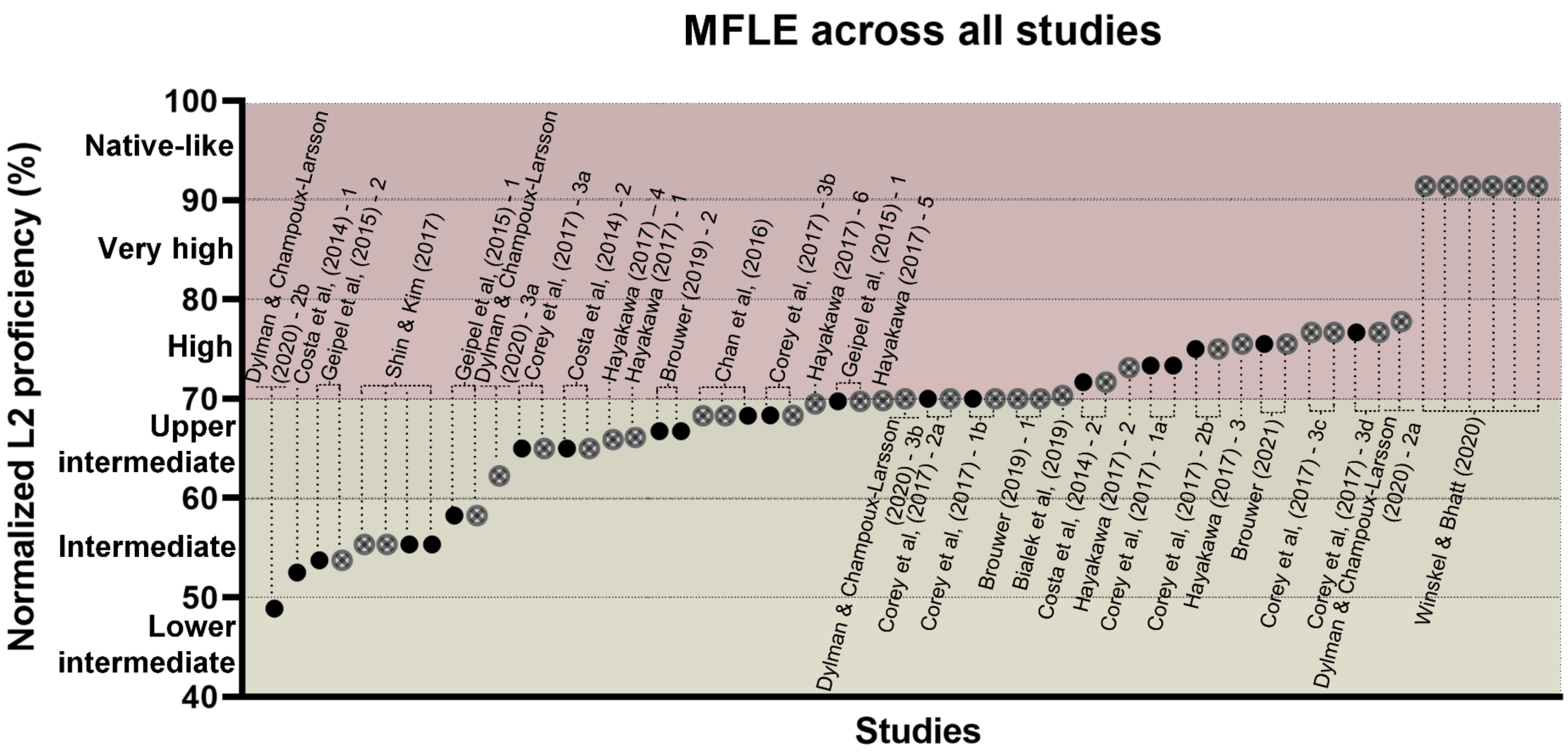

Figure S1. Moral foreign-language effect (MFLE) on moral dilemmas across all eligible studies. Studies are sorted from left to right on the X axis based on their samples' normalized L2 proficiency level. Black circles denote significant MFLEs. Crossed circles denote non-significant MFLEs.
